# Chromatin context shapes DNA damage formation and nucleotide excision repair dynamics in *Caenorhabditis elegans*

**DOI:** 10.1093/nar/gkaf1080

**Published:** 2025-11-04

**Authors:** Cansu Kose, Cem Azgari, Laura A Lindsey-Boltz, Ogün Adebali, Aziz Sancar

**Affiliations:** Department of Biochemistry and Biophysics, University of North Carolina School of Medicine, Chapel Hill, NC 27599, United States; Faculty of Engineering and Natural Sciences, Sabanci University, Istanbul, 34956, Türkiye; Department of Biochemistry and Biophysics, University of North Carolina School of Medicine, Chapel Hill, NC 27599, United States; Department of Biochemistry and Biophysics, University of North Carolina School of Medicine, Chapel Hill, NC 27599, United States; Faculty of Engineering and Natural Sciences, Sabanci University, Istanbul, 34956, Türkiye; Department of Biochemistry and Biophysics, University of North Carolina School of Medicine, Chapel Hill, NC 27599, United States

## Abstract

DNA damage formation and repair are influenced by the genomic landscape, yet how chromatin and transcriptional activity shape these processes at a whole-organism scale remains incompletely understood. Using *Caenorhabditis elegans*, a widely used model organism to study DNA repair and related processes, we present comprehensive, time-course maps of ultraviolet-induced DNA damage and excision repair, revealing how chromatin context and transcription dictate the spatiotemporal patterns of damage and repair. Of the two repair pathways—global repair and transcription-coupled repair—global repair predominates, removing the majority of the lesions; and notably, (6–4) photoproducts are removed by transcription-coupled repair at an extent comparable to cyclobutane pyrimidine dimers, a feature not previously observed in animals. Integration of damage and repair profiles with chromatin features reveals that, despite non-uniform damage formation, repair efficiency is the primary determinant of residual damage. Finally, repair around accessible regions exhibit nucleosome-size periodicity, reflecting underlying nucleosome architecture. Together, these findings establish *C. elegans* as a valuable model organism for interrogating damage formation and repair within a chromatin context and reveal species-specific features that broaden our understanding of DNA repair mechanisms across metazoans.

## Graphical Abstract

**Graphical Abstract:**
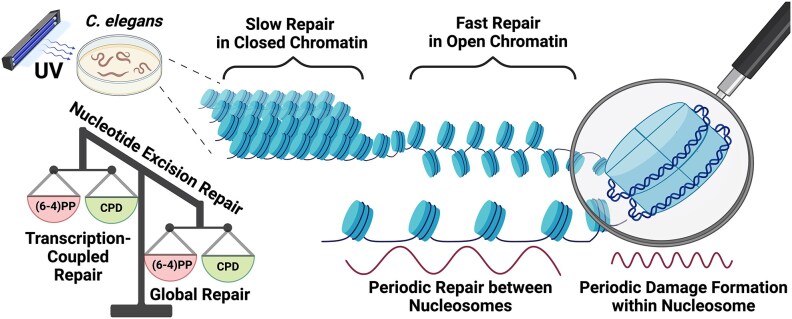

## Introduction

All living organisms, from bacteria to humans, must preserve genome integrity and have evolved mechanisms to repair DNA damage. Nucleotide excision repair (hereafter referred to as excision repair) is a critical pathway that removes bulky lesions such as ultraviolet (UV)-induced (6–4) photoproducts [(6–4)PPs] and cyclobutane pyrimidine dimers (CPDs). In humans, mutations in excision repair genes lead to a broad spectrum of symptoms, including UV sensitivity and increased cancer risk. The excision repair mechanism is highly conserved and proceeds through a stepwise process: (1) lesion recognition, (2) dual incisions flanking the damage, (3) release of the damaged oligonucleotide, and (4) repair synthesis and ligation [1]. Two distinct subpathways of excision repair have been characterized: global repair and transcription-coupled repair (TCR). In global repair, DNA lesions are detected by a set of damage-sensing factors, including XPC, RPA, XPA, and the TFIIH complex, which contains the XPB and XPD helicases [1, 2]. Once the damage is recognized, XPF and XPG endonucleases make incisions 5′ and 3′ to the damage, respectively, releasing a ∼26–30 nucleotide-long oligonucleotide containing the lesion [3–5]. DNA polymerase and ligase then complete the repair synthesis and sealing steps [6–8]. In TCR, RNA polymerase II (RNAPII) that has stalled at a lesion on the transcribed strand is recognized by CSB, CSA, and UVSSA. Displacement or degradation of RNAPII and other factors occur in coordination with TFIIH entry. This leads to the recruitment of XPA, RPA, XPG, and XPF to damage site, bypassing the need for XPC. The remaining steps, including dual incision and repair synthesis, mirror those of global repair [9–12].

UV radiation is a major environmental factor that induces DNA damage in living organisms. Upon absorption by DNA, UV photons directly trigger the formation of bulky photolesions, primarily CPDs and (6–4)PPs, which distort the DNA double helix and interfere with vital cellular processes such as replication and transcription. Although CPDs and (6–4)PPs are substrates of both excision repair pathways, the relative contribution of TCR to their processing varies among species. In humans and *Drosophila melanogaster*, TCR plays a significant role in the processing of CPDs, whereas removal of (6–4)PPs by TCR is not detectable unless global repair is perturbed [13, 14]. In contrast, *Arabidopsis thaliana* and *Saccharomyces cerevisiae* display prominent TCR of both CPDs and (6–4)PPs, although TCR of (6–4)PPs is markedly less [15, 16]. The relative difference in lesion processing is largely attributed to the greater DNA helix distortion caused by (6–4)PPs, which enhances their detection by global repair mechanisms, whereas CPD detection is facilitated by detection by RNAPII [17].

Our understanding of how DNA damage forms and is repaired in the context of the cellular environment, where DNA is constantly being transcribed, replicated, and packaged into higher order structures, remains incomplete. Although lesion formation itself is largely dictated by sequence and other chemical properties, prior studies have shown that the efficiency of excision repair varies considerably across chromatin landscapes [18–20]. For example, bulky DNA adducts tend to be repaired more rapidly in open chromatin regions, such as active promoters and strong enhancers, while repair is markedly slower in repressive or heterochromatic domains [19, 21–24]. Additionally, work in yeast, humans, and flies has demonstrated that rotational positioning of DNA around nucleosomes leads to preferential damage formation with ∼10 bp periodicity [24–26]. Yet, the nucleosome structure itself presents a physical barrier to repair, reducing incision efficiency and access to repair machinery.

Nucleotide excision repair has been studied in *Caenorhabditis elegans* since the 1980s [27, 28]. Most core components of the mammalian repair machinery are conserved in *C. elegans*, including XPA, XPB, XPC, XPD, XPF, XPG, CSB, CSA, and UVSSA [29–34]. However, the kinetics of UV-induced DNA damage repair in *C. elegans* differ from those observed in other metazoans [28]. Notably, CPDs and (6–4)PPs are removed with similar efficiency, unlike in humans where (6–4)PPs are removed much faster than CPDs [28, 35]. Supporting this observation, recent analyses of UV-induced mutational signatures in *C. elegans* have suggested that these signatures may be attributed to both CPDs and (6–4)PPs, consistent with their similar rates of repair [36]. Moreover, the contribution of excision repair to UV survival in *C. elegans* is developmentally regulated: global genome repair predominates in germ cells and embryos, whereas transcription-coupled repair becomes more critical for survival during larval and adult stages [32]. Despite these insights from mutational and survival studies, direct analysis of DNA repair activity in *C. elegans* has remained largely unexplored.

Here, we have generated high-resolution, genome-wide maps of (6–4)PP and CPD damage and repair in L1-stage *C. elegans* using two methods providing single nucleotide resolution called Damage-seq and eXcision Repair-seq (XR-seq), respectively. In short, Damage-seq is performed by capturing and sequencing sites where DNA polymerase stalls at damage lesions, whereas XR-seq is performed by capturing and sequencing the actual damage-containing oligos removed during excision repair. Unexpectedly, results from both methods revealed that (6–4)PPs and CPDs are repaired to similar extents by both TCR and global repair. Comparative profiling of damage and repair dynamics in wild-type, *csb-1* (TCR-deficient), and *xpc-1* (global repair-deficient) strains shows that global repair is the dominant repair pathway. Integration of the damage and repair maps with chromatin features, including H3K4me1, H3K4me3, H3K36me3, and H3K27me3, revealed that chromatin accessibility strongly predicts repair efficiency across all genotypes, albeit with distinct spatial patterns. While damage formation varies modestly across chromatin landscapes, we find that repair distribution more strongly determines residual damage. Finally, we observed a striking periodicity in early repair and late damage signals around strong ATAC-seq peaks, consistent with nucleosome phasing shaping the landscape of excision repair. Interestingly, while both CPDs and (6–4)PPs exhibit a 10 bp periodicity in damage formation within nucleosomal DNA, this pattern is retained during repair only for CPDs. In contrast, (6–4)PP repair lacks such periodicity, revealing a fundamental divergence, not only in how chromatin architecture modulates damage recognition and processing but also in how different photoproducts may differentially perturb nucleosome structure and accessibility. Together, our study positions *C. elegans* as a robust *in vivo* system for investigating excision repair within chromatin contexts and reveals species-specific features that refine our broader understanding of UV damage processing in metazoans.

## Materials and methods

### Biological resources

The *C. elegans* wild-type (N2), *csb-1* (RB1801), *xpc-1* (TG2226), and *xpa-1* (ok698) strains were obtained from the *Caenorhabditis* Genetics Center and were cultured under standard conditions at room temperature on nematode growth media (NGM) agar plates with *Escherichia coli* strain OP50.

### Excision assay

Excision assay performed as previously described [37]. Briefly, eggs were collected from adult animals by hypochlorite treatment and kept in M9 buffer at 22°C for 16 h with gentle rotation. Arrested L1 larvae were placed on NGM agar plates with OP50, fed for 3–4 h, then transferred to new NGM agar plates without bacteria and exposed to 4 kJ/m^2^ of UVB or 100 J/m^2^ UVC. After irradiation they were transferred to the plates with OP50. At the indicated repair times, worms are collected in and washed three times with M9 buffer and then, the pelleted worms were then incubated for 2 h at 62°C with 450 μl of Worm Hirt Lysis Buffer (0.15M Tris pH 8.5, 0.1M NaCl, 5mM EDTA, 1% SDS) and 20 μl of Proteinase K (NEB, cat. no. P8107S). Subsequently, 120 μl of 5M NaCl was added, and the mixture was inverted to ensure proper mixing, followed by an overnight incubation and 1 h centrifugation at 4°C. The supernatant was treated with Ribonuclease A (Sigma–Aldrich, cat. no. R4642) and Proteinase K. Next, DNA was precipitated using phenol-chloroform extraction and ethanol precipitation, dissolved in TE and incubated with anti-(6–4)PP (Cosmo Bio, cat. no. CAC-NM-DND-002) or anti-CPD (Cosmo Bio, cat. no. CAC-NM-DND-001) antibodies at 4°C overnight. Antibody-bound excised products were isolated, 5′ end (γ-[^32^P] ATP) radiolabeled and separated on 10 or 12% denaturing sequencing gel.

### XR-seq

Purified excised oligonucleotide containing either (6–4)PP or CPDs were processed for XR-seq assay, as described in [38, 39]. In brief, excised oligonucleotides were ligated to the adaptors, purified with the antibody used in the first purification, and DNA damage was reversed by either (6–4)PP or CPD photolyase. After PCR amplification, the library was sequenced with either Illumina HiSeq 4000 or NextSeq 2000 platforms.

### Damage-seq

L1 larvae were irradiated with 4 kJ/m^2^ UVB and collected in M9 buffer at indicated repair times. Worms were washed three times with M9 buffer and then genomic DNAs were isolated with QIamp DNA Mini Kit/tissue protocol and processed as described previously [21]. For naked DNA control Damage-seq, genomic DNA is irradiated after isolation from wild-type *C. elegans*. Ultrasonic fragmented genomic DNAs were purified using an equal volume of HighPrep PCR beads (MagBio). Purified DNA (∼1 µg) was used for end-repair and dA-tailing and adaptor ligation (NEBNext Ultra II DNA Library Prep Kit) following manufacturer’s instructions. Following purification of damage-containing DNA fragments with either anti-(6–4)PP (Cosmo Bio, cat. no. CAC-NM-DND-002) or Anti-CPD (Cosmo Bio, cat. no. CAC-NM-DND-001), the DNA was primer extended in the presence of 30 pmol Bio3U (biotin elongation primer. Next, undamaged DNA strands were captured by 20 pmol (2 µL) of SH oligo. DNAs were purified using phenol-chloroform extraction and ethanol precipitation. The DNA was then ligated to a second adaptor ligation using T4 DNA ligase HC (Thermo) at 16°C overnight. After quality check, the DNA samples were purified with HighPrep PCR beads, and ligated DNAs were PCR amplified by NEBNext Ultra II PCR Master Mix with NEBNext Multiplex Oligos for Illumina (New England Biolabs). The PCR libraries were purified with HighPrep PCR beads. Libraries were sequenced by an Illumina NextSeq2000P2 with 50-bp paired-end read.

### RNA-seq

Total RNA extracted from *C. elegans* with the protocol described in [40]. Briefly, L1 stage wild-type, *csb-1* and *xpc-1 C. elegans* were collected in M9 and washed until the supernatant was clear, followed by incubation with TRIzol and chloroform. After centrifugation at 14 000 g for 15 min at 4°C, the aqueous phase was mixed with an equal volume of isopropanol. Following centrifugation, the RNA pellet was washed several times and then resuspended in RNase-free water. Quality control, followed by stranded and poly(A) enriched library preparation and sequencing, was performed by Novogene.

### Slot blot

L1 larvae were irradiated with 75 J/m^2^ of UVB in NGM agar plates without bacteria. After treatment, worms were transferred back to the plates with *E. coli* and collected in M9 buffer at indicated time points. Worms were washed with M9 buffer three times. Genomic DNAs were isolated with Qiamp DNA Mini Kit/tissue protocol. For (6–4)PP slot blot 250 ng and for CPD slot blot 150 ng of DNA for each sample were loaded into a well of a slot blot apparatus. DNA was transferred to a membrane the membrane was dried in a vacuum oven at 80°C for 90 min and then blocked at room temperature for 1 h with 5% milk in 1X PBS with 0.1% Tween (PBS-T). Later, the membrane was washed with PBS-T three times, 5 min each, and then incubated with anti-CPD (Cosmo Bio Co., Ltd; NM-DND-001) or anti-(6–4)PP (Cosmo Bio Co., Ltd; NM-DND-002) antibodies at 4°C overnight. Membranes were washed with PBS-T as described above, and then incubated in secondary antibody, Rabbit-anti-mouse IgG (Abcam, cat. No. ab46540), at room temperature for 1 h. After washing as described above, membranes were developed using the Bio-Rad Western ECL Kit and imaged using the Bio-Rad ChemiDoc™ MP Imaging System. Following three washes in PBS-T, membranes were incubated with SYBR Gold (Thermo Fisher) at a 1:10 000 dilution in PBS-T to visualize and quantify total DNA loaded in each lane. Band intensities were quantified using Image Lab Software v6.1.0 (Bio-Rad). For each sample, the signal was first normalized to the SYBR Gold signal to account for DNA loading, and then further normalized to the initial damage. Statistical analyses and plotting were performed in GraphPad Prism 10 using one-way ANOVA with Geisser-Greenhouse correction, followed by Tukey’s multiple comparisons test.

## Bioinformatic analyses

### Adaptor trimming, removal of PCR duplicates, and alignment

XR-seq reads were trimmed to remove adaptor sequences by Cutadapt [41], and then duplicated reads were removed by fastx_toolkit / 0.0.14 (hannonlab.cshl.edu/fastx_toolkit/index.html). Trimmed reads were aligned to the C. *elegans* (ce11 ENSEMBL (Wbcel235, Gen Bank assembly accession: GCA_000002985.3) genomes using Bowtie2 with arguments -f -very-sensitive [42].

We only kept reads that: (i) have mapping quality greater than 20; (ii) are from chromosome I, II, III, IV, V, and X; and (iii) are of length 21–28 nt for downstream analysis after plotting nucleotide distribution and read length distribution of all mapped reads. Two biological replicates of each sample were merged for the following analysis.

For Damage-seq, reads containing the adaptor sequence GACTGGTTCCAATTGAAAGTGCTCTTCCGATCT were removed, as they are from undamaged strand, with cutadapt –discard-trimmed. Remaining reads were aligned with Bowtie2 with parameters -q –phred33 –local -p 4 –seed 123 –no-mixed to the *C. elegans* genome (ce11). For each sample, duplicated reads were reduced to a single read. Damage sites were identified as the two nucleotides upstream of each fragment. Using custom scripts, reads were trimmed to the first 4 nucleotides from the 5′ end and slopped 6 nucleotides upstream to generate 10 nucleotide-long genomic regions with the damaged nucleotide in the middle (Supplementary Fig. 1E–H). For the downstream analysis, reads with dipyrimidines at the expected damage sites were selected, and two biological replicates were merged.

For RNA-seq, reads were aligned to the *C. elegans* genome using STAR with –outFilterIntronMotifs RemoveNoncanonicalUnannotated [43]. Following alignment, only high-confidence, properly paired reads were retained. Specifically, SAM files were filtered to include reads that: (1) had a mapping quality (MAPQ) score ≥30, (2) were mapped as proper pairs on the same chromosome, (3) had an insert size ≤500 kb with correct orientation (→ ←), (4) contained a CIGAR string with at least one matching segment (i.e. including ‘M’), (5) mapped to ≤10 genomic locations (based on NH:i: tag), and (6) excluded any reads mapped to mitochondrial DNA (MT). Both soft- and hard-clipped reads were retained. Gene-level quantification was then performed using featureCounts with the WS295 *C. elegans* annotation [44].

### Damage-seq and XR-seq simulation

To generate expected background distributions for repair and damage maps, we used Boquila (v0.6.045), a next-generation sequencing read simulator designed to preserve nucleotide composition [45]. Boquila randomly selects genomic positions to generate pseudo-reads that match the dinucleotide (*k*-mer = 2) frequency and sequence complexity of the input dataset. Simulated datasets were generated separately for Damage-seq and XR-seq using the following parameters: –sens 20 –kmer 2.The resulting synthetic reads reflect the expected distribution of sequencing signal based solely on underlying sequence context and dimer frequency, allowing us to control for sequence composition when interpreting observed damage and repair enrichments genome wide.

### Genome-wide clustering of XR-seq signal in 2 kb windows

To assess genome-wide similarity in repair activity, we divided the *C. elegans* genome into non-overlapping 2 kb bins using bedtools makewindows [46] and intersected these bins with XR-seq read alignments using bedtools intersect RPM (reads per million) was calculated by normalizing the raw bin counts to the total mapped reads for that sample. The resulting RPM matrix was used to calculate pairwise Spearman correlation coefficients between all samples. A distance matrix (1 – Spearman correlation) was computed, and hierarchical clustering was performed using Ward’s method. Heatmaps were generated using the pheatmap R package.

### Repair profiles of TS and NTS

For plotting strand-based average repair profiles of the genes, we used WormBase WS295 genome annotations and filtered 2142 genes longer than 2 kb, situated at least 500 bp away from neighboring genes. For each gene, the region spanning from 1 kb upstream of the TSS to 500 bp downstream was divided into 100 bins. Similarly, the region from 1 kb upstream to 500 bp downstream of the transcription end site (TES) was also divided into 100 bins, resulting in a total of 200 bins per gene. Bed files of the reads were intersected to the 100 bin-divided-gene list by Bedtools intersect with the following commands -c -wa -F 0.5 -S or -s for TS and NTS, respectively.

For each *C. elegans* strain, RNA-seq data were used to calculate expression levels in terms of TPM, and genes were filtered to exclude those with no expression. The remaining genes were ranked by expression level and divided into four quartiles (low: 0–25%, medium-low: 25–50%, medium-high: 50–75%, and high: 75–100%), yielding 1 148 genes per quartile in wild-type, 1156 in *csb-1*, and 1161 in *xpc-1*. To analyze strand-specific damage and repair, we intersected Damage-seq and XR-seq data with these gene lists. For each gene, read counts were normalized to RPKM using the formula: RPKM = (number of reads / gene length in kb) / (total mapped reads / 1 million).

For each expression quartile, paired RPKM values for TS and NTS were compared using a two-sided Wilcoxon signed-rank test. Violin plots overlaid with boxplots were generated to visualize the distribution of RPKM values for TS and NTS across expression quartiles. For distribution of Damage-seq and XR-seq reads around TSS, 150 binned gene lists per quartile (or top half and bottom half) were generated to intersect with Damage-seq and XR-seq, as explained above.

### Epigenome Data Processing and Visualization

To investigate the influence of chromatin context on DNA damage formation and excision repair, we analyzed XR-seq and Damage-seq signals in the context of histone modification and chromatin accessibility data. Publicly available ChIP-seq datasets for H3K4me1, H3K4me3, H3K27me3, and H3K36me3 in L1 larval developmental stage were retrieved from GEO accession GSE114440 [47].Sequencing reads (FASTQ files) were aligned to the *C. elegans* reference genome using Bowtie2 with the –very-sensitive option. Aligned reads were converted to sorted BAM files with Samtools [48], and PCR duplicates were removed using samtools markdup -r. For each histone mark, biological replicates were merged using samtools merge.

To quantify the relationship between chromatin context and DNA damage/repair activity, we divided the C. elegans ce11 genome into consecutive non-overlapping 2 kb bins using the UCSC tool bedtools makewindows (via the UCSC utilities suite; e.g. bigWigAverageOverBed to extract per-bin scores from bigWig tracks). For each histone modification (H3K4me1, H3K4me3, H3K27me3, H3K36me3) as well as XR-seq, Damage-seq, and corresponding simulations, mean signal values were computed per 2 kb bin and written to .tab files. Histone bins were then ranked by signal intensity (mean0 column of the .tab output) after trimming the top 0.5% to reduce outlier influence, and split into three equal groups (low, medium, and high). These tertile labels were used to stratify DNA damage and repair signals relative to underlying chromatin context. Identical binning and tertile definitions were applied across Damage-seq, XR-seq, and their matched simulations to enable direct comparisons. Normalized XR-seq and Damage-seq values were divided by their respective simulation values (real/sim), log₂-transformed, and plotted as boxplots across tertiles for each time point and genotype. Significance testing was performed using paired Wilcoxon tests across time courses and unpaired Wilcoxon tests between tertiles, with Benjamini–Hochberg correction for multiple testing. Final plots were rendered with custom R scripts built on data.table and ggplot2, preserving consistent whisker definitions (12.5th–87.5th percentiles) and shared y-axis scales for wild-type and *csb-1*, with *xpc-1* scaled separately.

For functional integration with XR-seq and Damage-seq data with the ChIP-seq of histone markers, we performed peak calling with MACS2. For H3K4me1 and H3K4me3, we used –nomodel –extsize 147 to call narrow peaks. For H3K27me3 and H3K36me3, –broad and –broad-cutoff 0.1 were used to call broad peaks. In addition, for a second H3K36me3 dataset, we directly used the pre-processed peak BED file (GSM4275264_MACS2_N2_rep1_broad_Peaks.bed.gz) under GEO accession number GSM4275264 [49]. The number of peaks called was 22 032 for H3K4me1, 10 888 for H3K4me3, 10 954 for H3K36me3, and 13 107 for H3K27me3. 5 kb windows were centered on ChIP-seq peaks and divided into 500 bins (10 bp each). These binned regions intersected with strand-specific XR-seq and Damage-seq data using bedtools intersect -c. The resulting bed files captured read counts per bin per strand for each time point and histone mark.

For Damage-seq, raw read counts were first normalized to total mapped reads per strand to obtain RPM values. Each time point was then normalized to the corresponding mock-treated control, followed by normalization to the mean of the naked DNA control. The resulting profiles were corrected by dividing by simulation-derived signals, smoothed with an 11-bin rolling window, and transformed to log₂ scale. For XR-seq, per-bin read counts were similarly normalized to total mapped reads (RPM) and corrected by dividing by the corresponding simulation-derived profiles. Smoothed log₂-transformed signals with confidence intervals were generated for each damage type (CPD or (6–4)PP), time point, and genotype (wild-type, *csb-1, xpc-1*). To account for strand orientation, the bin order of the plus strand was reversed (e.g. the 500th bin plotted as the 1st), while the minus strand order was kept unchanged, ensuring transcription is consistently displayed from left to right. All plots were produced using custom Python scripts with Matplotlib.

To visualize enrichment patterns of histone marks relative to genes, peak-centered heatmaps were generated using deepTools [50]. Binary bigwig files were first created by converting peak regions to a 1-valued bedGraph format and transforming them into bigwig format using bedGraphToBigWig. Then, computeMatrix was run in scale-regions mode (–beforeRegionStartLength 500, –regionBodyLength 1000, –afterRegionStartLength 500, –binSize 10) using gene body annotations. Heatmaps were rendered with plotHeatmap function of deepTools.

### Repair profiles of open chromatin regions

For Fig. 5 A-F, ATAC-seq peaks defined by Jänes *et al.* specific to L1 larval developmental stage were first mapped from ce10 to ce11 by liftOver tool of UCSC genome browser [51]. Peaks were further categories into quartiles according to enrichment score. ATAC-seq peaks were also divided into genic (including 200 bp upstream of TSS) and intergenic peaks by intersecting with WS295 genome annotations. Peaks were slopped 500 bp upstream, 500 bp downstream and divided into 40 bins. Reads from XR-seq and Damage-seq intersected with the following commands -c -wa -F 0.5. Plots were generated by a custom R script.

ATAC-seq peaks quartiles were analyzed to assess their association with gene expression levels. Each quartile intersected with gene expression data obtained from wild-type *C. elegans* RNA-seq, which provided transcripts per million (TPM) values for each gene. The TPM values associated with genes overlapping the quartile-stratified ATAC-seq peaks were analyzed to assess the correlation between chromatin accessibility and gene expression. Each data point represents an individual gene’s expression level (TPM value) for the corresponding ATAC-seq peak intensity category.

### Calling ATAC-seq peaks and nucleosome dyads

Raw ATAC-seq reads were first processed to remove duplicated reads and residual adapter sequences using fastp [52] with the options –dedup –detect_adapter_for_pe. Deduplicated reads were aligned to the C. elegans ce11 reference genome using bowtie2 with the parameters –local –very-sensitive –no-mixed –no-discordant, specifying a minimum fragment length of 25 bp and a maximum of 700 bp. Alignments to the mitochondrial chromosome (chrM), reads with mapping quality below 30, unmapped or unpaired reads, and potential PCR duplicates were removed using samtools. Blacklisted regions defined for the ce11 genome [53] were excluded using bedtools. High-quality peaks were then called from the filtered BAM files using MACS2 [54], shifting reads upstream by 37 bp and extending them to a total of 73 bp, with significant peaks defined at FDR < 0.05. Summits identified by MACS2 were used to define precise ATAC-seq peak centers for downstream analyses. To identify nucleosome dyad positions, we applied nucleoATAC [55] with default parameters, which infers nucleosome positions from ATAC-seq BAM files by modeling fragment length distributions and Tn5 transposase insertion sites. In total, 19 651 high-confidence ATAC-seq peak regions and 9149 nucleosome dyad centers were identified for downstream analyses.

For downstream analyses, regions were binned to generate aggregated profiles. For ATAC-seq peaks in Fig. 5G and H, we used 2 kb windows centered on peak summits (±1 kb) divided into 201 bins of 10 bp each. For nucleosome dyads in Fig. 6, we used 160 bp windows centered on the dyad position (±80 bp) divided into 41 bins of 2 bp each.

### Quantification of nucleosome periodicities

Calculations of the power spectrum and SNR were performed using the approaches adapted from the methodology de- scribed in Pich *et al.* [56]. Briefly, to calculate power spectrum, we mean-centered binned signal and computed power at a defined range of candidate periods using a Fourier-based approach. For each target period P, we calculated the squared magnitude of the sum of signal values weighted by complex exponentials, effectively measuring how strongly the signal matched a repeating pattern of that period. This yielded a power value for each tested period, normalized by the length of the signal. To achieve comparable values across timepoints and genotypes, the resulting power values were further normalized by dividing by their means, producing a dimensionless measure of relative periodicity strength. Defining only a group of candidate periods allowed us to focus specifically on biologically relevant periodicities, such as ∼160 bp corresponding to nucleosome spacing or ∼10 bp corresponding to DNA helical turns around nucleosome dyads. For analyses of *xpc-1* mutant signals, power spectrum calculations were restricted to the downstream portion of the region, starting from basepair position + 250 for ATAC-seq-centered analyses and + 20 for dyad-centered analyses, to specifically capture transcription-driven asymmetry.

### Quantification of Signal-to-noise Ratio

To assess the robustness of the detected periodicity, we calculated the signal-to-noise ratio (SNR) for each region, defined as the ratio of the power at the target periodicity (160 bp for nucleosomes; 10 bp for dyad-centered analyses) to the mean power across all other frequencies. This provided a normalized measure of periodic signal strength relative to background noise. To evaluate statistical significance, we employed a permutation-based approach to derive empirical *P*-values. For each region, repair and damage signals were randomly shuffled 1000 times, with the SNR recalculated for each permutation to generate a null distribution. The empirical *P*-value was then calculated as the proportion of permuted SNR values exceeding the observed SNR.

## Results

### Capture and characterization of DNA damage-containing excised oligonucleotides from *C. elegans*

**Figure 1.**
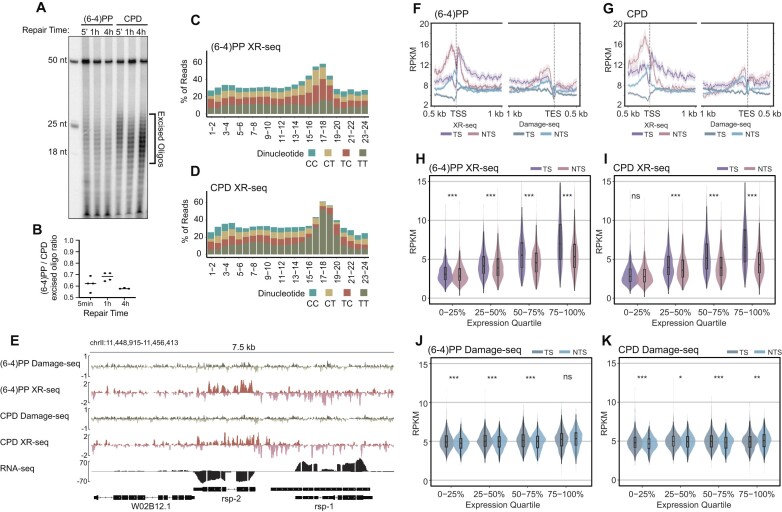
Genome-wide mapping of excised oligos revealed transcription-coupled repair of (6–4)PP. (**A**) Denaturing gel showing excised oligonucleotides containing (6–4)PP and CPD lesions collected at 5 min (5′), 1 h, and 4 h after UVB treatment (4 kJ/m²). The first lane contains 18-, 25-, and 50-nt size markers; the 50-mer was included as an internal labeling control in each lane. Brackets indicate the excised product range. (**B**) Quantification of the excision assay signal shown in (A), from four biological replicates, plotted as mean ± s.e.m. (**C**) Dipyrimidine composition along 24-nt XR-seq reads 1 h post-UV exposure reveals enrichment of TC dinucleotides at position − 6 (from the 3′ end) in (6–4)PP XR-seq and TT dinucleotides in (**D**) CPD XR-seq, indicating damage specificity. (**E**) Genome browser screenshot of strand-separated Damage-seq at 0 h and XR-seq at 1 h read coverage over a 7.5-kb region on chromosome II. The bottom track shows RNA-seq signal to indicate transcriptional orientation of *rsp-2* and *rsp-1*. XR-seq reads show strand bias toward the transcribed strand, whereas Damage-seq reads exhibit no strand bias at 0 h. Repair strand asymmetry is observed in transcribed regions, and the three genes in this locus display varied expressions: W02B12.1 (low), rsp-2 (high, minus strand), and rsp-1 (high, plus strand). The transcribed strand (TS)/ non-transcribed strand (NTS) repair ratios for (6–4)PPs in rsp-2 and rsp-1 were ∼3.17 and ∼3.49, respectively, whereas damage formation ratios were ∼0.93 and ∼1.18, indicating TCR of (6–4)PP. CPD repair TS/NTS ratios were similarly enriched at ∼4.92 (rsp-2) and ∼3.5 (rsp-1), with damage formation ratios of ∼0.78 and ∼1.2. (**F, G**) Metaprofiles of RPKM-normalized XR-seq (pink/purple) and Damage-seq (light/dark blue) signal for TS and NTS around 2142 genes (>2 kb, non-overlapping, ≥500-bp spacing). Plots span 500 bp upstream to 1 kb downstream of the TSS and 1 kb upstream to 500 bp downstream of the TES. Shaded regions represent 95% confidence intervals. (**H–K**) Violin plots showing strand-specific quantification of XR-seq (**H, I**) and Damage-seq (**J, K**) signal for (6–4)PPs and CPDs in the gene bodies across four gene expression quartiles (based on RNA-seq). Boxplots indicate the median and interquartile range. Paired Wilcoxon signed-rank tests were used to compare TS and NTS values; asterisks denote significance: ***P < 0.001, **P < 0.01, *P < 0.05, ns = not significant.

To investigate excision repair in *C. elegans*, we irradiated L1-stage worms with 4 kJ/m^2^ UVB, allowed time for repair, and then captured the damage-containing excised oligonucleotides by immunoprecipitation with damage-specific antibodies against either (6–4)PP or CPD. Excised fragments were isolated at 5 min, 1 h, and 4 h post-UV treatment, and then analyzed after radiolabeling and gel electrophoresis (Fig. 1A). For both damage types, the earliest time point, 5 min, captured the longest excision products, ranging from 16 to 30 nucleotides (nt) and peaking at ∼24 nt. The oligonucleotides were likely partially degraded during the 1- and 4-h timepoints as the observed peak fragment sizes shifted down to 22 and 21 nt, respectively. The striking difference between the two damage types was their relative abundance. Using a sequential antibody pulldown strategy from the same lysates, we determined that the (6–4)PP/CPD ratio of excised products ranged approximately between 0.5 and 0.7. (Fig. 1B). Considering that UVB induces CPDs at ∼8-fold higher frequency than (6–4)PPs, these excision ratios indicate that (6–4)PPs are excised at a frequency ∼4-fold higher than CPDs [57, 58].

Next, we constructed XR-seq libraries from the excised oligonucleotides containing the two different damage types captured 1 h after UVB (4 kJ/m^2^) to determine the genome-wide locations of the repair events. Analysis of the (6–4)PP and CPD XR-seq read lengths showed a distribution between 10 and 32 nt with a peak at 24 nt (Supplementary Fig. 1 A and B), consistent with the excision assay results. Analysis of the DNA base composition along each position of the 24 nt-long reads revealed pyrimidine enrichment of TC, CT, and TT dinucleotides 6 nt from the 3′ end in (6–4)PP libraries (Fig. 1C) and TT enrichment pattern for CPD libraries (Fig. 1D), in agreement with previous studies in other organisms [13–15]. Although XR-seq reads longer than 28 nt accounted for less than 5% of the total, nucleotide frequency plots of reads up to 30 nt-long still showed thymine and cytosine enrichment at 7 and 8 nt from the 3′ end of reads for (6–4)PP (Supplementary Fig. 1C) and thymine enrichment at the same position for CPD (Supplementary Fig. 1D). The dipyrimidine enrichment pattern was not seen in the reads longer than 30 nt indicating that this is the maximum size oligonucleotide removed by nucleotide excision repair in *C. elegans*.

### Transcription-coupled repair plays a significant role in the removal of both (6-4)PPs and CPDs in L1-stage *C. elegans*

XR-seq reads were mapped to the *C. elegans* genome and a 7.5 kb genome browser view illustrates the similar non-uniformity in repair of both (6–4)PP and CPD damages (Fig. 1E). Strand asymmetries are observed in the transcribed regions of the three genes in this locus which display varied levels of expression: W02B12.1 (low), rsp-2 (high, minus strand), and rsp-1 (high, plus strand). More (6–4)PP and CPD repair reads map to the transcribed strand (TS) than the non-transcribed strand (NTS) of the genes undergoing transcription, which is the hallmark of TCR. To assess whether damage formation was responsible for the repair strand-specificity, we performed genome-wide Damage-seq immediately following UV in L1-stage *C. elegans*. Similar to previous reports, our Damage-seq results show enrichment of dipyrimidines at the expected damage site (Supplementary Fig. 1E–H) [21]. As illustrated in the genome browser view, the Damage-seq signals for both damages are relatively uniform and thus the strand asymmetry is due to repair (Fig. 1E). We next generated metaprofiles of Damage-seq and XR-seq signal across transcription start sites (TSS) and transcription end sites (TES) of 2142 selected genes. Both (6–4)PP and CPD repair signals showed clear strand bias favoring the TS within gene bodies (Fig. 1F and G). We observed elevated repair on the NTS upstream of TSSs and TESs; however, after normalizing to the simulation to account for sequence bias, this strand-specific repair difference disappeared and reversed, in the upstream of the TSS and TES, respectively (Supplementary Fig. 1I and J). Nevertheless, for both strands XR-seq signals in the upstream of TSSs are relatively higher than expected based on Damage-seq RPKM values, indicating that repair activity exceeds the baseline predicted by damage formation alone.

To evaluate the transcriptional dependence of repair strand bias, we stratified genes into quartiles based on RNA-seq expression and quantified stranded (based on transcription direction) RPKM values for both XR-seq and Damage-seq data. XR-seq revealed robust transcription-dependent repair for both (6–4)PPs and CPDs with repair signals increasing with gene expression on both strands but were much more pronounced on the transcribed strand especially in the top two expression quartiles (Fig. 1H and I). Notably, repair also increased on the NTS, suggesting that global repair is enhanced at highly expressed genes, possibly due to greater chromatin accessibility. For Damage-seq, we observed modest but statistically significant differences in TS versus NTS damage levels, with NTS having more damage in the top quartile (Fig. 1J and K). However, these differences did not scale with expression level and were largely attributable to sequence composition, as validated by simulations (Supplementary Fig. 1K and L). Taken together, this transcription-dependent asymmetry further confirms that TCR contributes to the repair of (6–4)PPs, like CPDs, in *C. elegans*.

### Dominance of global repair revealed through repair kinetics and read distributions across the genomes of wild-type and repair-deficient strains

**Figure 2.**
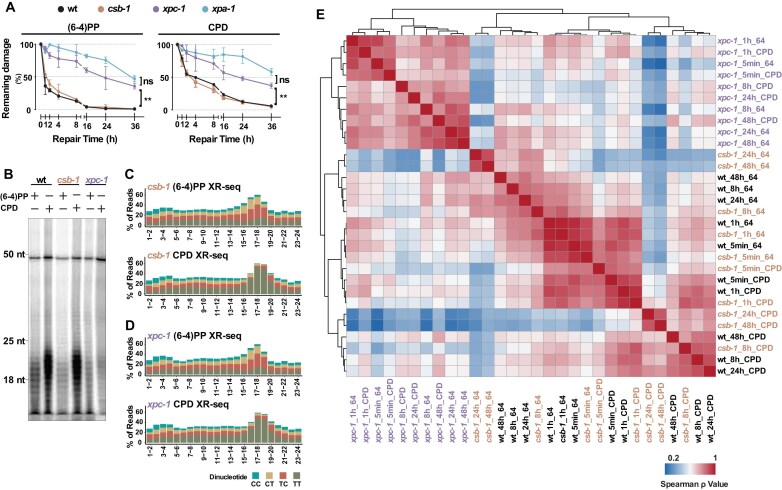
Repair kinetics and genomic distribution of repair events are similar between wild-type and *csb-1*, but distinct from *xpc-1*. (**A**) Quantification of (6–4)PP and CPD removal over time in wild-type (wt), *csb-1, xpc-1*, and *xpa-1*. Genomic DNA was isolated at the indicated time points after UVB treatment (75 J/m^2^) and probed with an anti-(6–4)PP (left) and an anti-CPD antibody (right). Damage signals were normalized to time 0 and plotted as mean ± s.e.m. from ≥3 biological replicates. Statistically significant differences between genotypes reflect impaired repair in *xpc-1* and *xpa-1* mutants relative to wt. No significant differences were observed between wt and *csb-1* (not shown). Statistical comparisons were performed using one-way ANOVA with Geisser-Greenhouse correction followed by Tukey’s multiple comparisons test. Asterisks denote significance: **P < 0.01, ns = not significant. (**B**) Denaturing gel showing excised oligonucleotides containing (6–4)PP and CPD collected 2 h after UVC treatment (100 J/m²) in wt, *csb-1*, and *xpc-1* strains. Size markers (18, 25, and 50 nucleotides) are indicated on the left. A 50-mer internal labeling control was included in each lane. (**C**) Dinucleotide composition of 24-nt XR-seq reads for (6–4)PP and CPD in *csb-1*. (**D**) Corresponding dinucleotide composition for (6–4)PP and CPD in *xpc-1*. (**E**) Hierarchical clustering heatmap of pairwise Spearman correlations between XR-seq samples from wt, *csb-1*, and *xpc-1* across the UV repair time course. The heatmap is based on RPM mapped reads from 2 kb genomic windows. Clustering reflects the similarity in genome-wide repair signal distributions between genotypes and time points.

We employed the following strains of *C. elegans* to tease apart the contributions of TCR and global repair to the dynamics of damage and repair: *csb-1* (TCR-deficient), *xpc-1* (global repair-deficient), and *xpa-1* (excision repair-deficient). First, we monitored the kinetics of (6–4)PP and CPD removal from the genomes of these strains using a slot blot assay which quantitatively measures the amount of DNA damage remaining in genomic DNA at different timepoints after UV irradiation (Fig. 2A). To enable sensitive detection of early repair events without saturation, we applied a moderate dose of UVB, 75 J/m^2^. The DNA damage was detected using lesion-specific monoclonal antibodies and normalized to total DNA with subsequent staining with SYBR gold (Supplementary Fig. 2A and B). In wild-type animals, (6–4)PPs and CPDs were almost completely removed by 24 h and 36 h, respectively. Notably, >50% of (6–4)PPs were excised within 1 h, whereas CPDs reached ∼50% repair by 2–4 h, consistent with the ∼4-fold faster repair of (6–4)PPs seen with the excision assay in Fig. 1A. Interestingly, *csb-1* mutants exhibited nearly identical repair kinetics to wild-type for both lesions, indicating that TCR contributes minimally to overall excision activity. By contrast, *xpc-1* mutants showed substantially impaired repair, similar to *xpa-1* mutants. The modest decrease in signal seen in *xpa-1* over time is not due to repair activity but rather reflects passive dilution of unrepaired damage through cell division and growth. This interpretation is supported by our previous study, which showed that *xpa-1* lacks detectable repair above background [38]. Together, these findings indicate that global repair is the primary pathway responsible for removing UV-induced photoproducts in L1-stage *C. elegans*.

Next, we captured the excised oligos containing either (6–4)PPs or CPDs from wild-type and mutant strains 1 h after UV irradiation. As shown in Fig. 2B, the excision pattern in *csb-1* was similar to that of the wild-type, whereas significantly fewer excised oligonucleotides were recovered from the *xpc-1* strain, which lacks global repair, despite using six times more worms. XR-seq was performed with captured oligos and the nucleotide distribution analysis confirmed comparable dipyrimidine content for both (6–4)PP and CPD from the *csb-1* and *xpc-1 strains* (Fig. 2C and D), as was seen for wild-type samples.

In order to measure the dynamics of repair we collected excised oligos at five different timepoints following UV irradiation (5 min, 1 h, 8 h, 24 h, and 48 h). We performed hierarchical clustering using Spearman correlations of the time-course XR-seq data (Fig. 2E). The samples separated into two major clusters: *xpc-1* formed a distinct group, while wild-type and *csb-1* clustered together. Within the *xpc-1* cluster, (6–4)PP and CPD damage are grouped by timepoint (early vs. late), rather than by damage type, reflecting the loss of damage-specific excision in the absence of global repair and that both 6–4(PP)s and CPDs are repaired with a similar genomic distributions by TCR. In contrast, wild-type and *csb-1* samples resolved into three distinct clusters reflecting both time and damage type: (1) (6–4)PPs at late timepoints, (2) early timepoints for both damage types, and (3) CPDs at late timepoints. This pattern was preserved when biological replicates were analyzed independently (Supplementary Fig. 3). Together, the slot blot and genome-wide XR-seq analyses consistently show that wild-type and *csb-1* animals exhibit highly similar excision repair profiles, whereas *xpc-1* displays globally impaired repair with distinct genome-wide distribution of repair events.

### Transcription profoundly shapes excision repair dynamics in *C. elegans*

**Figure 3.**
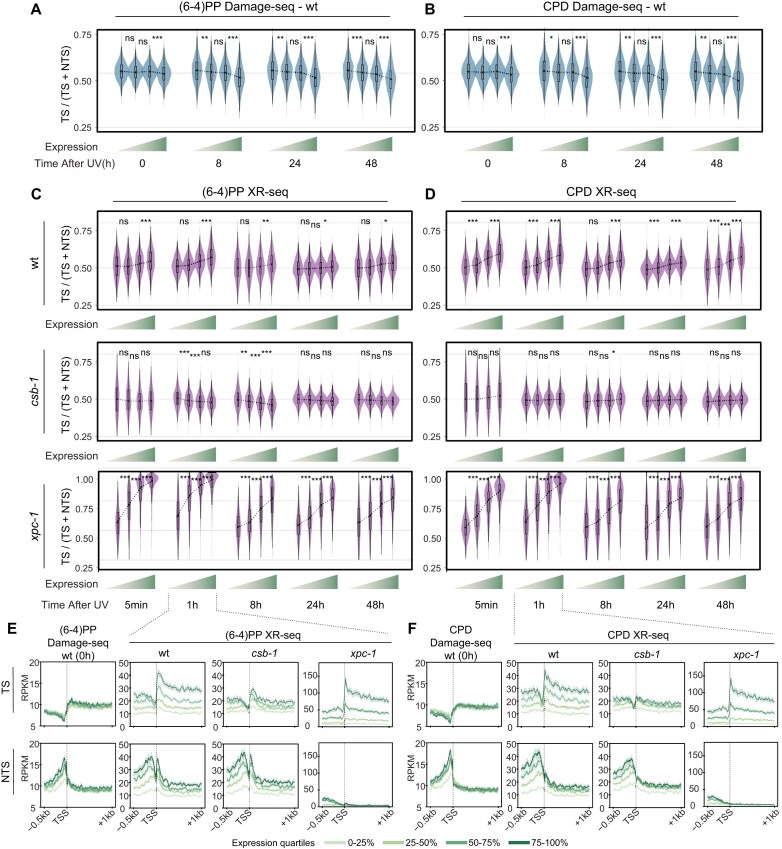
Expression-dependent dynamics of UV-induced DNA damage and repair in wild-type, *csb-1*, and *xpc-1*. (**A**) Violin plots showing the ratio of transcribed strand signal over total signal (TS / [TS + NTS]) across gene expression quartiles for (6–4)PP and (**B**) CPD Damage-seq data from wild-type (wt) at 0, 8, 24, and 48 h post-UV. Subsequent rows show XR-seq data from wt, *csb-1*, and *xpc-1* at 5 min, 1 h, 8 h, 24 h, and 48 h post-UV for (**C**) (6–4)PP and (**D**) CPD. Boxplots within violins show the median and interquartile range. Genes were stratified into expression quartiles based on each strain’s RNA-seq TPM values, increasing left to right (0–25%, 25–50%, 50–75%, 75–100%), indicated by a green gradient below the x-axis. Statistical comparisons between adjacent quartiles were performed using two-sided Wilcoxon signed-rank tests. Asterisks indicate significance: ***P < 0.001; **P < 0.01; *P < 0.05; ns = not significant. (**E**) Metaprofiles of Damage-seq (0 h) and XR-seq (1 h post-UV) signal across TSS for (6–4)PP and (**F**) CPD. Signal is plotted separately for the TS (top) and NTS (bottom) across a region spanning 500 bp upstream to 1 kb downstream of the TSS. Genes are divided into quartiles based on RNA-seq expression. Line colors indicate increasing expression from light green (lowest quartile) to dark green (highest quartile); shaded areas represent 95% confidence intervals.

To investigate how transcription influences excision repair dynamics over time, we analyzed strand-specific Damage-seq and XR-seq signals across gene expression quartiles in the wild-type and repair-deficient strains (Fig. 3A–D). The wild-type XR-seq analysis revealed strand-biased repair at all time points (5 min to 48-h post-UV) (Fig. 3C, D, top panel). Correspondingly, Damage-seq results from the wild-type showed that the amount of remaining damage in the TS of highly expressed genes was significantly lower at all time points post-UV (8 h, 24 h, and 48 h) compared to initial damage formation indicated at 0 h (Fig. 3A and B). In XR-seq from the *csb-1* strain, strand bias was lost for CPDs at all time points, consistent with the lack of TCR (Fig. 3C and D, middle panel). For (6–4)PPs, strand bias was inverted at 1 h and 8 h, with more NTS repair than TS, suggesting transcription-dependent inhibition of repair on the transcribed strand in the absence of the CSB transcription-coupled repair factor as previously reported for *E. coli* and human cell lines [59, 60]. As we previously reported, *xpc-1* repair was strongly biased toward the TS at all time points and across all expression quartiles (Fig. 3C and D, bottom panel) [39].

To further examine the distribution of damage and repair events across the TSS, we analyzed strand-specific metaprofiles of Damage-seq at 0 h and XR-seq at 1-h post-UV, stratified by expression quartiles (Fig. 3E and F). For damage formation, one notable expression-dependent feature was reduced NTS signal upstream of the TSS in low-expression quartiles for both (6–4)PP and CPD. This observation is recapitulated in sequence-based simulation models (Supplementary Fig. 4A and B), indicating sequence content dependent damage formation. In wild-type, both TS and NTS repair increased with expression level, with a stronger effect on the TS. Notably, repair upstream of the TSS also scaled with expression, in contrast to the distribution in the simulation (Supplementary Fig. 4A and B), suggesting additional contributing factors such as bidirectional promoter activity or enhancer RNA transcription occurring on the same strand as the downstream gene [39, 61]. In *csb-1*, repair increased with expression on both strands; however, no preferential repair of the TS was observed within gene bodies, consistent with the absence of transcription-coupled repair. Nonetheless, *csb-1* showed an increase in both TS and NTS repair correlating with expression levels upstream of the TSS, consistent with enhanced repair in open chromatin regions [23]. In *xpc-1*, repair was almost exclusively restricted to the TS across all expression quartiles. TS repair scaled with expression, while NTS repair remained negligible. TS repair upstream of the TSS also correlated with expression, though to a lesser extent than in gene bodies. The preferential TS repair upstream of the TSS likely supports the productive elongation of enhancer RNAs on the same strand as the downstream gene [39, 61].

We next examined repair dynamics across time around the TSS of highly expressed genes (Supplementary Fig. 4C). For both (6–4)PPs and CPDs, Damage-seq revealed a decrease in TS signal, consistent with preferential TS repair. In wild-type, XR-seq showed a pronounced peak of TS repair at 1 h, particularly near the TSS, reflecting a window of efficient transcription-coupled repair. Previous studies have shown that dissociation of RNAPII from damaged bases, followed by transcription restart at the TSS, promotes earlier repair of the TS at the 5′ end of genes [62]. Our time-course data in both wild-type and *xpc-1* are consistent with this model. In contrast, *csb-1* did not display a TS repair peak at 1 h. Analysis of the bottom half of expressed genes (Supplementary Fig. 4D) further showed that repair occurred predominantly at later time points across all strains, underscoring the influence of transcription level on the timing of repair.

### Chromatin context shapes both damage formation and the dynamics of excision repair in *C. elegans*

**Figure 4.**
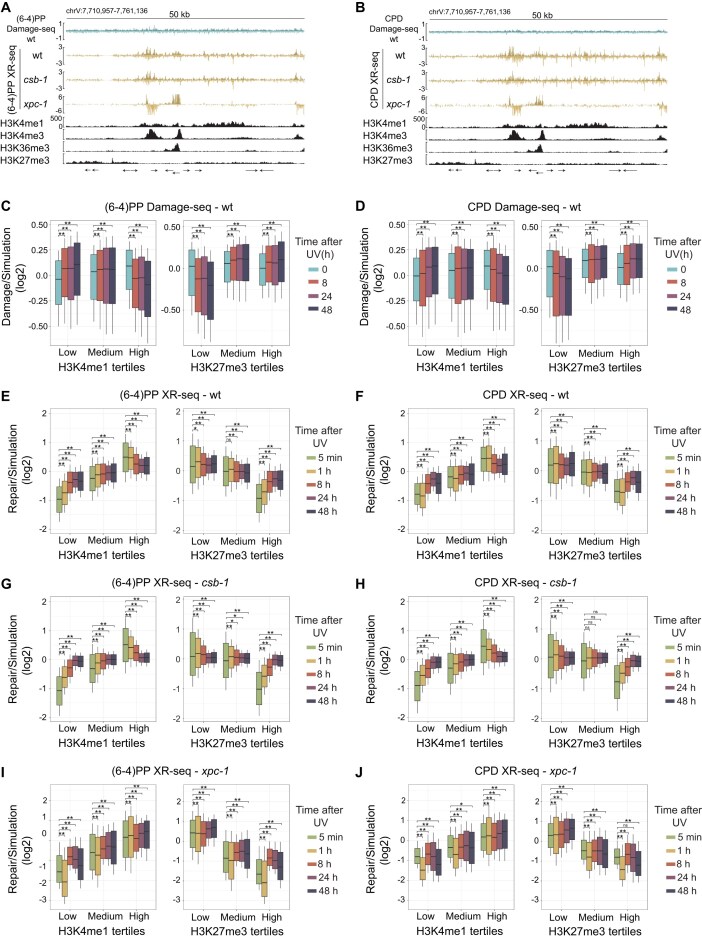
Influence of histone modifications on UV-induced DNA damage formation and repair. (**A**) Genome browser tracks showing stranded Damage-seq (top row, 0 h), XR-seq repair activity (1 h) in wild-type (wt), *csb-1*, and *xpc-1*, and ChIP-seq signals for histone modifications H3K4me1, H3K4me3, H3K36me3, and H3K27me3 across a representative 50 kb region on chromosome V. Damage-seq and XR-seq tracks are shown for (6–4)PP and (**B**) CPD. Arrows at the bottom indicate the orientation of genes longer than 2 kb. (**C**–**J**) Boxplots showing log₂-normalized Damage-seq (**C, D**) and XR-seq (**E–J**) signals divided by simulation profiles, grouped by tertiles of H3K4me1 (left panels) or H3K27me3 (right panels) signal in 2 kb bins. Data are shown for wt (**E, F**), *csb-1* (**G, H**), and *xpc-1* (**I, J**). Time points correspond to 0–48 h after UV irradiation for Damage-seq (C, D) and 5 min–48 h for XR-seq (E–J). Significance was determined by paired Wilcoxon tests relative to 5 min (within time course) and unpaired Wilcoxon tests between tertiles, with Benjamini–Hochberg correction. Stars denote adjusted *P*-values: ns, not significant (*P* ≥ 0.05); * *P* < 0.05; ** *P* < 0.01.

Previous studies have shown that active and open chromatin regions were repaired more rapidly than other genomic regions [23]. Here, we evaluated damage formation and time-course repair in active (marked by H3K4me1, H3K4me3, and H3K36me3) and repressed genomic regions (marked by H3K27me3) by using ChIP-seq datasets from Jänes *et al.*, 2018. In a representative 50-kb window on chromosome V, Damage-seq signals were relatively uniform, while XR-seq at 1-h post-UV showed increased repair in regions enriched for H3K4me1, H3K4me3, and H3K36me3, and reduced signal over H3K27me3 peaks (Fig. 4A and B).

For genome-wide quantification, we stratified regions into low, medium, and high enrichment levels based on the signal intensity of each histone modification. Initial Damage-seq profiles showed similar overall damage across categories, but decay patterns revealed less remaining damage in highly H3K4me1-enriched active regions and more remaining damage in strongly H3K27me3-enriched repressed regions, a trend consistent for both (6–4)PPs and CPDs (Fig. 4C and D). Complementary to this, XR-seq demonstrated that regions with high H3K4me1 enrichment repaired earlier, whereas strongly H3K27me3-enriched regions repaired later in wild-type and *csb-1* (Fig. 4E–H). In *xpc-1*, repair in active regions peaked at 1 h, while repressed regions exhibited reduced repair at the same time point, consistent with the transcription-coupled repair peak observed at 1 h (Fig. 3C). Similar trends to those observed for H3K4me1 were also seen for the other active marks, H3K4me3 and H3K36me3 (Supplementary Fig. 5).

We also examined stranded damage and repair signals centered on ChIP-seq peaks of the four histone modifications (Supplementary Fig. 6A–D). Interestingly, we found that damage formation was not uniform across peak centers and flanking regions: for example, (6–4)PPs showed distinct peak formation at the centers of H3K4me1, H3K36me3, and H3K27me3, whereas CPDs exhibited weaker enrichment. The distribution of time-course damage signals revealed that at late time points, damage decreased at the centers of active mark peaks, whereas the opposite trend was observed for the repressive mark. Complementarily, repair signals were strongly enriched at the centers of H3K4me1 and H3K4me3 peaks in wild-type and *csb-1*, while in *xpc-1* repair was asymmetrically enriched on one side of the peaks, reflecting transcriptional directionality. Repair around H3K36me3 displayed distinct patterns: in wild-type and *csb-1* enrichment was stronger in flanking regions. In *xpc-1* repair was biased toward the opposite side of the peaks, consistent with H3K36me3 marking elongation and supporting inversion of TCR polarity relative to promoter regions[47, 63]. To further investigate repair directionality, we analyzed a second H3K36me3 dataset from Carpenter *et al.* (2021) [49]. Surprisingly, in this dataset, repair signals in *xpc-1* were symmetrically distributed around H3K36me3-enriched regions (Supplementary Fig. 6E). Whereas dataset from Jänes *et al.*, 2018 showed broad enrichment across gene bodies with stronger signal near TESs than TSSs, the Carpenter dataset showed the reverse pattern, with strong enrichment near TSSs and depletion near TESs (Supplementary Fig. 6F). These discrepancies between the datasets likely reflect variation in antibody choice across studies.

Collectively, these data demonstrate that the presence of histone modifications affects both the location and timing of UV-induced lesion formation and repair. Promoter- and enhancer-associated marks (H3K4me1, H3K4me3) are associated with early and transcriptionally oriented repair. Gene body H3K36me3 regions display delayed repair with inverted transcription-coupled repair polarity. Polycomb-repressed regions marked by H3K27me3 exhibit limited repair. These findings support a dual role for chromatin context in shaping genome integrity by influencing both damage susceptibility and repair efficiency.

### Chromatin accessibility impacts damage formation and excision repair dynamics

**Figure 5.**
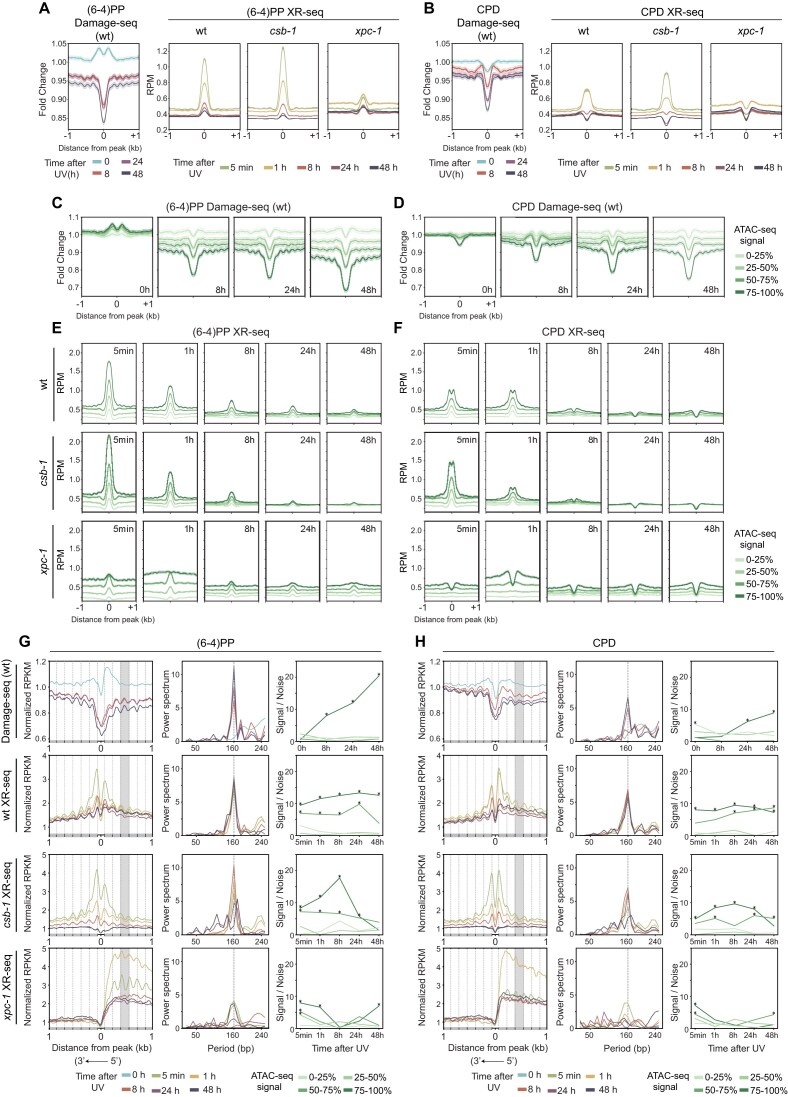
Time-course analysis of UV-induced damage and repair signal at ATAC-seq peaks. (**A**) Line plots showing Damage-seq (left) and XR-seq (right) signals around ATAC-seq peaks for (6–4)PP and (**B**) CPD. Damage-seq data are shown at 0 h, 8 h, 24 h, and 48 h post-UV, normalized to both input and naked DNA controls and plotted as fold change. XR-seq signals are plotted as RPM for wt*, csb-1*, and *xpc-1* across 5 min, 1 h, 8 h, 24 h, and 48 h post-UV. (**C**) Damage-seq fold-change signal for (6–4)PP and (**D**) CPD is shown across the same time points, with ATAC-seq peaks stratified into four quartiles by chromatin accessibility (enrichment signal). Curves from light to dark green correspond to increasing ATAC-seq signal (0–25%, 25–50%, 50–75%, and 75–100%). (**E**) XR-seq RPM signal for (6–4)PP and (**F**) CPD is plotted over the same peak groups and time course for wt, *csb-1*, and *xpc-1*. All panels span ± 1 kb from the ATAC-seq peak center. Data plotted with 95% confidence intervals in (A-F), indicated in shaded regions around the lines. (**G**) Simulation-normalized Damage-seq (top) and simulation- and damage-normalized XR-seq signals for (6–4)PP and (**H**) CPD plotted across time points, centered on the most accessible quartile of L1-stage ATAC-seq peaks. For visualization, the bin order on the plus strand was reversed to align the transcriptional direction of both strands. Gray bars below each plot indicate nucleosome-wrapped regions, and black bars indicate linker DNA positions. Highlighted gray areas show damage and repair profiles at the third nucleosome position. (G, H, middle, right) Power spectrum and signal-to-noise ratio (SNR) plots quantifying 160-bp periodicity in Damage-seq and XR-seq signals across genotypes and time points. Stars denote statistical significance; * *P* < 0.05.

To investigate the influence of chromatin accessibility on UV damage and repair dynamics, we analyzed time-course Damage-seq and XR-seq signals centered on ATAC-seq peaks (Fig. 5). First, we plotted signals without considering strand orientation in the analysis. Damage-seq signal at 0 h revealed a distinct dip at the center of ATAC-seq peaks, flanked by enriched signal in the immediate ±200-bp regions for (6–4)PP (Fig. 5A). Simulated reads, however, did not exhibit the dip at the peak centers (Supplementary Fig. 7A and B). Therefore, the reduced damage formation precisely at ATAC-seq peak centers suggests the inhibitory effect of DNA-binding proteins on damage formation rather than a sequence bias (Fig. 5A and B) [19, 21]. Damage signal decreases by time for both damages as being more prominent in (6–4)PP. The repair signal, on the other hand, generally peaked at early time points and declined gradually over time (Fig. 5A and B, right panels). For (6–4)PPs, wild-type and *csb-1* showed strong repair enrichment at 5 min. CPD repair levels in wild-type were similar at 5 min and 1 h, with a gradual decrease with time. In *xpc-1*, both damage types showed maximal repair at 1 h in accessible regions, consistent with transcription-coupled repair being most active at this timepoint. We next categorized ATAC-seq peaks as genic or intergenic and observed that both were repaired at similar rates in wild-type and *csb-1* (Supplementary Fig. 8). In *xpc-1*, although early repair signals were more evenly distributed and elevated in genic regions, CPD repair at intergenic peaks became comparable to that at genic peaks by later time points. This pattern suggests transcription-dependent repair activity in intergenic regions and supports our previous observation of transcriptional activity in these regions, as detected by XR-seq in *xpc-1* [39].

Since ATAC-seq was performed on whole animals, the observed accessibility reflects an aggregate of diverse cell types. To identify sites that are consistently accessible across most nuclei, we stratified ATAC-seq peaks into four quartiles based on their enrichment scores. The top quartile represents the highest accessibility, allowing us to focus on regions likely to be constitutively open. In Fig. 5C and D, we plotted time-course Damage-seq signals across these quartiles and observed the strongest damage formation biases in the highest-accessibility group, a pattern not attributable to sequence composition (Supplementary Fig. 7C and D). Damage levels in the top two accessibility quartiles decreased steadily over time, revealing a stable periodicity pattern centered around ATAC-seq summits, most prominently in the highest-accessibility quartile.

XR-seq signals further highlighted the relationship between chromatin accessibility and repair efficiency. Across all time points and for both damage types, the most accessible ATAC-seq peaks (top quartile) consistently exhibited the highest repair activity in wild-type and *csb-1* animals (Fig. 5E and F). Due to preferential early repair, signal intensity declined over time but remained elevated in these regions, suggesting that global repair operates more efficiently in highly accessible chromatin. These patterns were absent in simulations (Supplementary Fig. 7E and F), supporting their biological relevance. Notably, the strongest quartile separation in *xpc-1* was observed at 1 h, aligning with the time of maximal TCR activity as shown in Fig. 3E and F. This is consistent with our observation that chromatin accessibility correlates with transcriptional activity (Supplementary Fig. 7G) [64].

We next analyzed whether the periodic signal patterns observed around accessible regions were driven by nucleosome positioning. To address this, we analyzed raw ATAC-seq data to generate four quartiles by accessibility score (Fig. 5G and H). Using the top quartile of peaks, we plotted Damage-seq and XR-seq profiles time-course. We normalized the Damage-seq by simulation; the XR-seq by the simulation-normalized damage levels to reflect repair efficiency relative to the local damage landscape (raw data and simulation-normalized data plotted in Supplementary Fig. 9). To more accurately capture directional differences in chromatin organization around peak centers, we reoriented the plus-strand Damage-seq and XR-seq signals. This was particularly important for resolving repair periodicity in *xpc-1* mutants, where transcription-coupled repair introduces strand-specific asymmetry relative to gene orientation. In wild-type animals, both (6–4)PP and CPD Damage-seq signals showed a periodic pattern at all time points after damage formation (Fig. 5G and H, top). At initial time point (0 h), damage levels were elevated overall without showing a detectable periodicity, while by 24 and 48 h the periodic peaks became more prominent. This time-dependent sharpening of the periodic pattern reflects preferential repair of linker DNA regions, leaving unrepaired lesions increasingly enriched in nucleosome-wrapped DNA. Consistently, XR-seq profiles in wild-type and *csb-1* exhibited strong periodic patterns at early time points (5 min, 1 h), with clear peaks that aligned closely with nucleosome repeat lengths (Fig. 5G and H, middle). The periodicity of repair around nucleosomes gradually diminished over time. In *xpc-1*, we also observed clear periodicity at early time points (5 min and 1 h in (6–4)PP, 5 min in CPD); however, this pattern was restricted mainly to the downstream of the peak centers, suggesting that transcription-coupled repair is also shaped by nucleosome occupancy. Moreover, the slight shift in periodic peaks from linkers in *xpc-1* may reflect increased nucleosome remodeling dynamics in the transcribed regions.

To quantitatively assess the presence and strength of periodic patterns in Damage-seq and XR-seq signals, we used a Fourier-based approach to calculate power spectrum at defined period lengths centered on ATAC-seq peak summits allowing us to detect and characterize dominant spatial frequencies in the data, such as those corresponding to nucleosome periodicity. We evaluated the signal-to-noise ratio (SNR) of these periodic components to ensure that observed periodicity reflected genuine biological phasing rather than random fluctuations across the quartiles of ATAC-seq summits. Analysis of the resulting power spectra with a peak periodicity at ∼160-bp and SNR plots (Fig. 5G and H, right) confirmed an increasing periodicity of damage over time and consistent periodic repair, aligned with nucleosome spacing in the highly accesible regions. In contrast, XR-seq signals for wild-type and *csb-1* animals exhibited strong ∼160-bp periodicity with high SNR at early time points (5 min, 1 h), which remained relatively stable over time despite declining overall repair signal intensity. For XR-seq of *xpc-1* mutants, we calculated periodicity specifically in the downstream region of peak centers, reflecting the transcription-driven orientation of repair in these samples. Although overall values were lower compared to wild-type and *csb-1*, both the power spectra and SNR at early time points remained elevated and significant for the most accessible peaks.

Together, our data demonstrate that at accessible chromatin regions, the extent of damage formation does not solely predict the level of residual damage. Instead, both TCR and global repair efficiency, driven by distinct underlying mechanisms, emerge as the primary determinants of damage removal. Chromatin accessibility promotes efficient repair by facilitating either pathway, reinforcing the idea that repair outcome is shaped more by repair dynamics than by initial damage deposition.

### Nucleosome-centered repair periodicity differs between (6-4)PPs and CPDs

**Figure 6.**
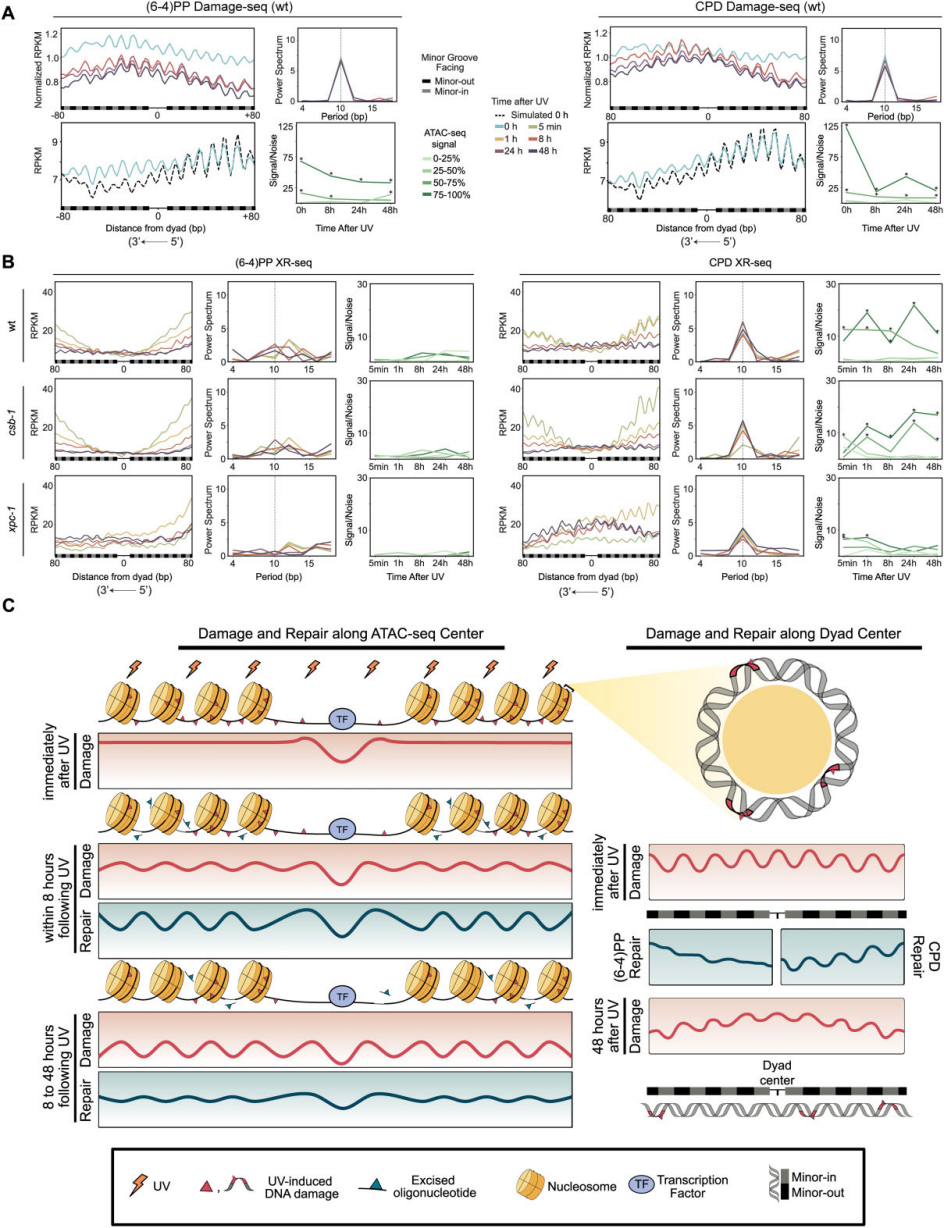
Periodicity of UV-induced damage and repair signals around nucleosome dyad centers. (**A**) Damage-seq signals and periodicity analysis for (6–4)PP (left group) and CPD (right group). Within each group, the top left panel shows simulation-normalized Damage-seq RPKM profiles at 0, 8, 24, and 48 h post-UV, while the bottom left panel shows Damage-seq and sequence-based simulated Damage-seq data at 0 h as RPKM values. Corresponding power spectrum (top, right) and SNR (bottom, right) plots quantify 10 bp periodicity in simulation-normalized Damage-seq signals across time points. (**B**) XR-seq signals are plotted as RPKM values for wt, *csb-1*, and *xpc-1* across 5 min, 1 h, 8 h, 24 h, and 48 h post-UV, with power spectrum and SNR plots quantifying 10 bp periodicity in XR-seq signals across time points for (6–4)PP (left) and CPD (right). Gray bars below each plot indicate minor-in positions, and black bars indicate minor-out positions. All panels span ± 80 bp from the dyad center. (**C**) Model illustrating the dynamics of damage formation and repair around nucleosomes and dyad centers. The left panel illustrates how UV-induced damage initially forms broadly across nucleosomes but develops periodic patterns over time as preferential repair of linker regions leaves remaining damage enriched on nucleosomes. The right panel zooms in on the nucleosome dyad, showing how DNA wrapping creates ∼10 bp periodicity in simulation-normalized damage distribution aligned with minor groove orientation. Although there is random repair of (6–4)PPs and periodic repair of CPDs, damage levels decrease overall over the time course, the damage periodicity remains persistent.

We next investigated periodicity in damage and repair distribution around nucleosome dyad centers (Fig. 6A and B). For both (6–4)PPs and CPDs, Damage-seq profiles centered on nucleosome dyads displayed clear ∼10 bp periodicity across all time points, reflecting the structure of DNA wrapping around the histone core. We showed that the raw damage signals consistently peaked at minor-in positions, where the DNA minor groove faces inward toward the histone surface (Fig. 6A, for both lesion types, RPKM). However, when simulation-normalized, damage levels shifted to peak at minor-out positions, indicating higher relative damage accumulation where the minor groove faces outward (Fig. 6A, for both lesion types, simulation-normalized RPKM). This shift reflects that while TT dinucleotide content peaks at minor-in regions (Supplementary Fig. 10A), the excess of damage relative to sequence expectation is higher at minor-out positions, consistent with previous reports showing CPD formation preferentially on minor-out after correcting for sequence composition [25, 26, 56].

Importantly, the ∼10 bp periodicity was preserved across time points, even as overall damage levels declined, indicating a stable pattern driven by the minor groove orientation. Unlike the overall repair distribution, which showed a dip at the nucleosome dyad for both (6–4)PPs and CPDs in wild-type and *csb-1* strains, only CPD repair retained this ∼10 bp periodicity, while (6–4)PP repair did not (Fig. 6B). After normalizing to both simulation and damage formation/simulation (Supplementary Fig. 10B), this periodicity in CPD repair disappeared, demonstrating that the apparent repair pattern mirrors the highly periodic characteristic of damage formation rather than reflecting an intrinsic preference of the repair machinery for specific DNA orientations. Thus, CPD repair acts uniformly across the nucleosomal landscape, effectively balancing local variations in damage susceptibility [26].

## Discussion

Although nucleotide excision repair is conserved across eukaryotic organisms, the mechanisms of dual incision, kinetics of UV-induced photoproducts removal, and the involvement of TCR vary significantly among species [13–15, 65]. While *C. elegans* has been used to model human cancer mutational signatures [36, 66] and genetic diseases such as Cockayne syndrome [67], fully understanding its excision repair mechanism is crucial for accurate interpretation of the results. In mammalian cells, the primary excised oligonucleotides are typically 26–30 nt-long, but degrade rapidly, often being observed as shorter fragments bound by RPA with a median size of around 20 nt [4, 68]. Our study was performed on whole animals, and the results therefore reflect an aggregate of diverse cell types, with neurons and muscle cells comprising the majority. We found that in *C. elegans*, UV-induced photoproductss are excised with a median length of 24 nucleotides via dual incisions made 16 nt 5′ and 6 nt 3′ from the damage site. The excised oligonucleotides gradually degrade from 5′ without accumulating. The differences in dual incision sizes observed across species may reflect species-specific variations in oligonucleotide stability, the DNA unwinding bubble created by XPB and XPD helicase [69] or differences in incision sequence preferences of XPF [70].

Importantly, we discovered that (6–4)PPs are repaired by TCR to an extent comparable to CPDs in *C. elegans*. While yeast and plants show TCR of (6–4)PPs at a lesser extent compared to CPDs [15, 16], TCR-mediated repair of (6–4)PPs had not been demonstrated previously in wild-type animals, including human, fly, and lemur, prior to this study [13, 14, 71]. The slower removal rate of (6–4)PPs in *C. elegans* compared to humans might explain why (6–4)PPs are comparably repaired via TCR rather than global repair [35]. However, it remains unclear why (6–4)PPs are removed more slowly in *C. elegans* or, conversely, more rapidly in other organisms. To validate these findings, we attempted to conduct an *in vitro* excision assay using *C. elegans* nuclear extract and defined DNA substrates (data not shown). Despite testing multiple protocols, we did not observe detectable excision repair activity, possibly due to low levels of repair factors or elevated nuclease activity in the extracts [72, 73]. Further structural studies of global repair factors in *C. elegans* could elucidate determinants of repair efficiency.

Our slot blot analyses indicated that 50% of (6–4)PPs were repaired within 1 h, whereas 50% of CPDs were repaired in 2–4 h. Complete removal of (6–4)PPs occurred by 24 h, while CPDs took up to 36 h. These results differ from earlier findings showing similar repair kinetics for both photoproducts across *C. elegans* developmental stages [28]. Two factors likely contribute to this discrepancy: First, the earlier study used UVC radiation, whereas we used UVB. UV wavelength and dose significantly influence photoproduct formation; for UVB, the CPD/(6–4)PP ratio is about eight, whereas for UVC, it is approximately four [57, 58, 74]. Thus, our study had higher relative CPD formation, and we also used a low dose of UVB so that repair was not saturated. Second, differences in antibody sensitivity and specificity between studies may have impacted observed repair kinetics. Despite (6–4)PPs being repaired more rapidly than CPDs in our analysis, their repair rates remain much slower compared to human cells.

Our study included *csb-1*, a TCR-deficient strain, and *xpc-1*, a global repair-deficient strain to understand the contributions of both pathways to the repair of UV-induced photoproducts. We observed similar repair kinetics and genomic distribution of repair events in wild-type and *csb-1* L1 *C. elegans*, but repair in the *xpc-1* strain was severely compromised. This indicates that the contribution of TCR to the overall genomic repair is only minor, but TCR ensures that the highly transcribed genes are repaired early. Earlier studies on the involvement of global repair and TCR in the UV response across different developmental stages found that TCR is the main mechanism for UV resistance in the L1 stage [32]. Although these findings might seem contradictory to our studies, it is important to note that survival and repair are very different endpoints. While most of the damage is removed by global repair, the presence of intact TCR ensures that expressed genes continue to function, which is crucial for the survival and development of L1 stage *C. elegans*, consisting primarily of non-dividing somatic cells [75].

We have presented detailed time-course repair dynamics for the TSs and NTSs, revealing highly similar (6–4)PP and CPD repair patterns. Damage-seq demonstrated consistently lower TS damage at 8, 24, and 48 h post-UV, reflecting ongoing TCR activity even at later time points. Unlike in *Drosophila*, yeast, and human cells, we did not observe increased NTS repair over TS repair at late time points, possibly due to the high UV dose applied in our experiments [76]. Perhaps such strand-switching of repair would become apparent at even later times or with lower doses of UV. Repair distributions correlated strongly with transcriptional activity across all strains: TS repair in wild-type and *xpc-1* indicated TCR activity, whereas correlations observed on the NTS in wild-type and both strands in *csb-1* reflected accessibility and global repair efficiency. Intriguingly, over time, transcription negatively correlated with TS repair for (6–4)PPs in *csb-1*, supporting previous observations of transcriptional inhibition of repair via stalled RNAPII in the absence of CSB [59, 60]. Analysis of repair time-course strandedness highlighted global repair-dependent early repair upstream of TSS, consistent with promoter accessibility [61]. The highest TCR efficiency appeared around 1 h post-UV at the 5′ ends of genes on the one side of the peaks, possibly reflecting RNAPII release and re-initiation at promoters [62]. Despite documented antisense transcription upstream of promoters in *C. elegans*, we observed no preferential NTS repair upstream of TSS in *xpc-1* for both damages, consistent with our previous findings [14, 39, 61].

Previous studies have linked histone modifications to mutation distribution, implicating higher mutation rates in heterochromatin; however, correlations between histone modifications and damage formation have not been fully explored [77]. Leveraging *C. elegans*’ smaller genome and resulting deeper sequencing depth, our damage and repair profiles around ChIP-seq peaks (H3K4me1, H3K4me3, H3K36me3, and H3K27me3) revealed novel insights. Damage formation was particularly enriched at the 5′ side of peaks. Repair patterns, however, diverged from damage formation distributions. Wild-type and *csb-1* showed similar repair dynamics differing markedly from *xpc-1*, whose repair profile distinctly reflected active transcription-coupled repair. Regions marked by H3K4me1, H3K4me3, and H3K36me3 underwent earlier repair compared to genomic averages, whereas H3K27me3-marked regions demonstrated persistently lower repair efficiency and thus higher residual damage. Thus, our results demonstrated that chromatin context emerges as one of the key determinants of the excision repair in *C. elegans*. 

Multiple studies have shown that nucleosome positioning can inhibit nucleotide excision repair, reducing repair efficiency in nucleosome-wrapped DNA compared to more accessible linker regions [25, 78, 79]. By integrating accessible genomic regions, ATAC-seq peaks, with time-course damage and repair maps, we demonstrate that nucleotide excision repair in *C. elegans* indeed preferentially occurs in linker regions, while nucleosome-wrapped DNA exhibits slower and less efficient repair (Fig. 6C, left). In contrast, damage formation is initially uniform across nucleosomes, but a clear ∼160-bp periodicity emerges over time as preferential repair of linker DNA leads to the periodic removal of lesions. Relatively higher damage remaining in nucleosomes can contribute to elevated mutation rates, as shown in various studies reporting nucleosome periodicity in certain tumor types [56, 78-80]. Notably, skin melanoma exhibits increased somatic mutation density in nucleosome-wrapped DNA, which is consistent with our model. Importantly, both *csb-1* and *xpc-1* mutants displayed periodic repair profiles, indicating that nucleosome positioning constrains both global and transcription-coupled nucleotide excision repair pathways. Moreover, we have demonstrated this periodicity not only for CPD lesions but also for (6–4)PPs, indicating that impaired nucleosome repair in *C. elegans* is independent of the DNA damage type.

We have analyzed the dynamics of repair and damage across nucleosome dyad centers (Fig. 6C, right). Unlike the wider regions with periodic nucleosomes, within individual nucleosomes damage formation around the dyad center is not uniform. Although the TT-rich sequence context of minor grooves leads to higher levels of damage accumulation on the inward-facing side (minor-in) downstream of dyads, after sequence context normalization, we find that DNA facing outward (minor-out) accumulates elevated damage levels than expected, while minor-in DNA shows lower damage levels relative to its sequence potential, suggesting that the histone octamer may act as a physical barrier and DNA bending around the nucleosome could limit damage formation at inward-facing minor grooves, as proposed in a previous study [24-26]. For CPDs, we observed that repair profiles exhibited ∼10 bp periodicity when examined as raw RPKM values, mirroring the highly periodic distribution of damage around the nucleosome dyad center. When repair signals were normalized by both the sequence-based simulation and the actual damage levels, this periodicity disappeared, which suggests that the repair process itself does not have an intrinsic preference for minor-in or minor-out orientations but instead accumulates in proportion to the damage pattern, as also discussed for the yeast genome [24]. Notably, (6–4)PP repair does not show any periodicity around dyads with or without damage normalization, even though its damage profile remains highly periodic across time points. It is notable that, unlike CPD, the initial (6–4)PP repair profiles do not exhibit the periodicity characteristic of damage formation. The reason why there is such a difference between (6–4)PP and CPD might be due to the fact that their helix-distorting severance. (6–4)PP is more destabilizing than CPD, causing ∼44° bend in helix compared to mild distortion (∼9°) by CPD [17]. (6–4)PP formation with severe helix distortion might induce nucleosome repositioning, which might result in partial nucleosome unwrapping [81]. Such an unwrapping might cause nucleosome position shifts, which is the likely reason for the lack of damage-mirrored repair periodicity for (6–4)PP. For both (6–4)PP and CPD, we observed increasing repair signal intensity when moving away from the dyad center, which can be attributed to the easier access of repair proteins at nucleosome boundaries due to nucleosome “breathing” dynamics [26]. In this context, damage profiles at late time points inversely correlate with early repair profiles, showing higher remaining DNA lesions at the dyad center and lower levels at the flanks. In summary, we observed a periodic damage profile with a ∼10 bp spacing that corresponds to the orientation of the DNA minor groove which is in line with elevated UV-induced mutations at those sites [56].

Altogether, our work provides a high-resolution view of how chromatin context and transcription shape the landscape of UV-induced DNA damage and nucleotide excision repair in a multicellular animal. By integrating time-resolved maps of CPD and (6–4)PP damage and repair with chromatin accessibility and transcriptional features, we reveal that repair kinetics and the resulting persistence of DNA lesions are governed more by chromatin accessibility and repair activity than by damage formation itself. The prominent role of transcription-coupled repair in removing (6–4)PPs challenges long held assumptions about the efficient removal of this damage by global repair in animals. Furthermore, the observed link between repair periodicity and nucleosome organization underscores the influence of chromatin architecture on DNA damage and repair dynamics. Together, these findings advance our understanding of genome maintenance in metazoans and highlight the value of whole organism approaches for elucidating the spatial and temporal coordination of DNA repair.

## Supplementary Material

gkaf1080_Supplemental_File

## Data Availability

The data have been deposited in the Gene Expression Omnibus (GEO) of the National Center for Biotechnology Information (NCBI) under accession numbers GSE280347, GSE280348, and GSE280349. All codes for bioinformatic analysis and figure generation are deposited in Github (CompGenomeLab/worm-UV-DR) and Zenodo (https://doi.org/10.5281/zenodo.17162181).
