# Supplementary material for: Chromatin context shapes DNA damage formation and nucleotide excision repair dynamics in Caenorhabditis elegans
Source: Nucleic Acids Res. 2025 Nov 4;53(20):gkaf1080. doi: 10.1093/nar/gkaf1080 (PMC12585911; doi:10.1093/nar/gkaf1080)
Supplement: gkaf1080_Supplemental_File [file gkaf1080_supplemental_file.pdf]

Supplementary Figures

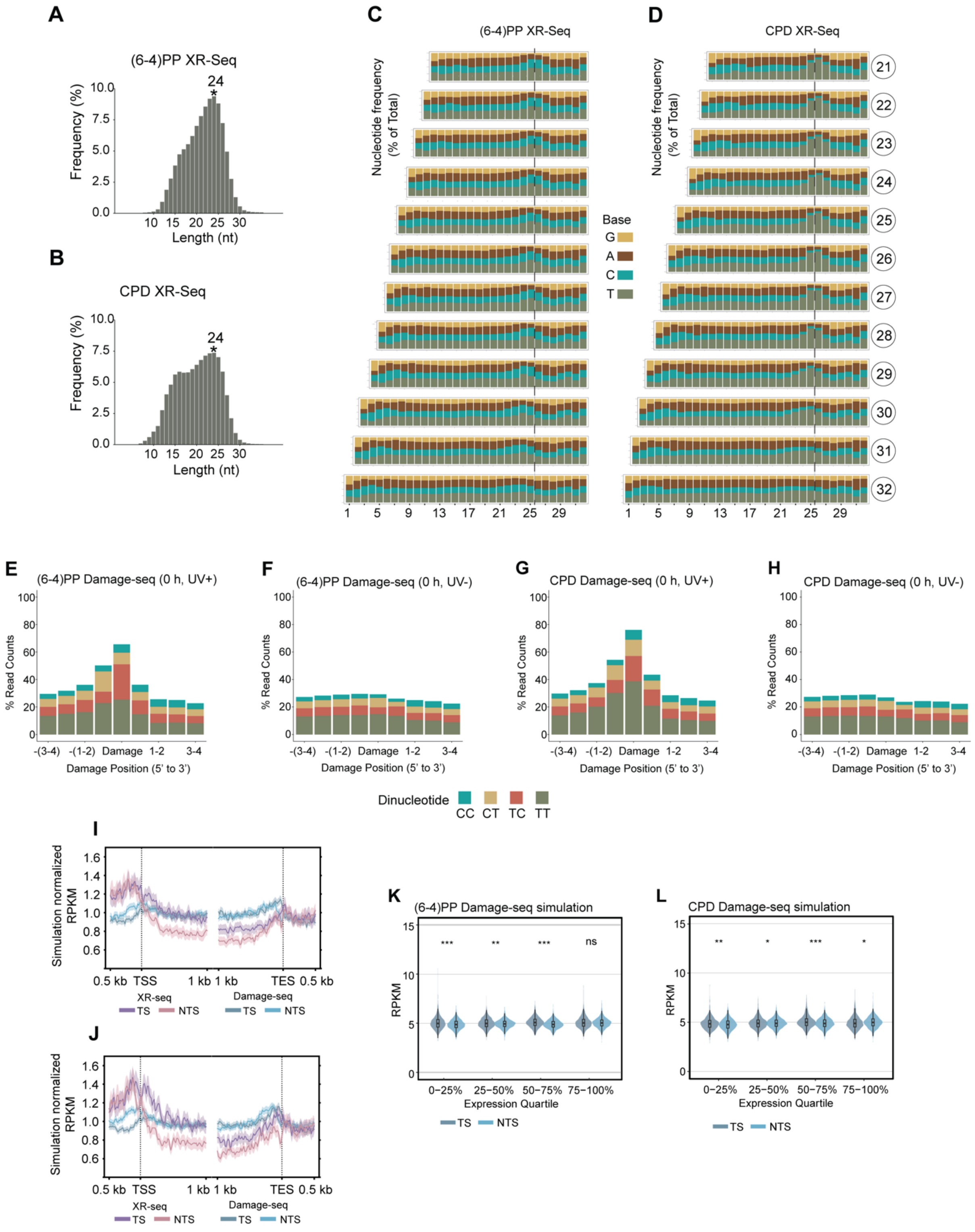

Supplementary Fig. 1: Characteristics of excised oligonucleotides and damage formation during nucleotide excision repair in *C. elegans*.

A, B Length distributions of excised oligonucleotides containing (6-4)PP (A) and CPD (B) from XR-seq data collected 1 h post-UV. **C, D** Nucleotide frequency profiles along 21–32 nt XR-seq reads for (6-4)PP (C) and CPD (D). **E**, **F** Dinucleotide distributions of (6-4)PP Damage-seq immediately post-UV (E) and mock-treated (F). **G, H** Dinucleotide distributions of CPD Damage-seq immediately post-UV (G) and mock-treated (H). **I, J** Simulation normalized Damage-seq and XR-seq data corresponding to main text Fig. 1f (I) and Fig. 1g (J), respectively. **K, L** Simulated Damage-seq data corresponding to main text Fig. 1i (K) and Fig. 1j (L), respectively.

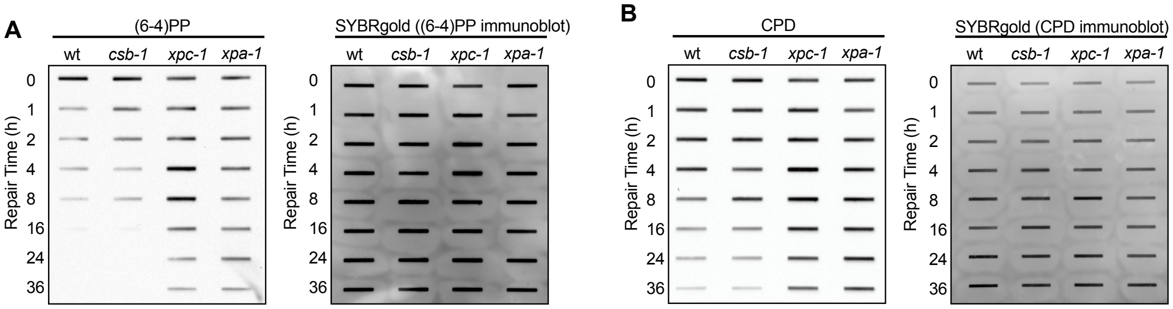

Supplementary Fig. 2: Representative images from slot blot experiments used in Fig. 2. A On the left, genomic DNA was isolated at the indicated time points after UV treatment and probed with an anti-(6-4)PP antibody. SYBRgold staining of slot blot membranes after (6-4)PP immunodetection in wild-type (wt), *csb-1*, *xpc-1*, and *xpa-1* across the indicated repair time points post-UV. **B** Same as in a, except the membranes were probed with an anti-CPD antibody.

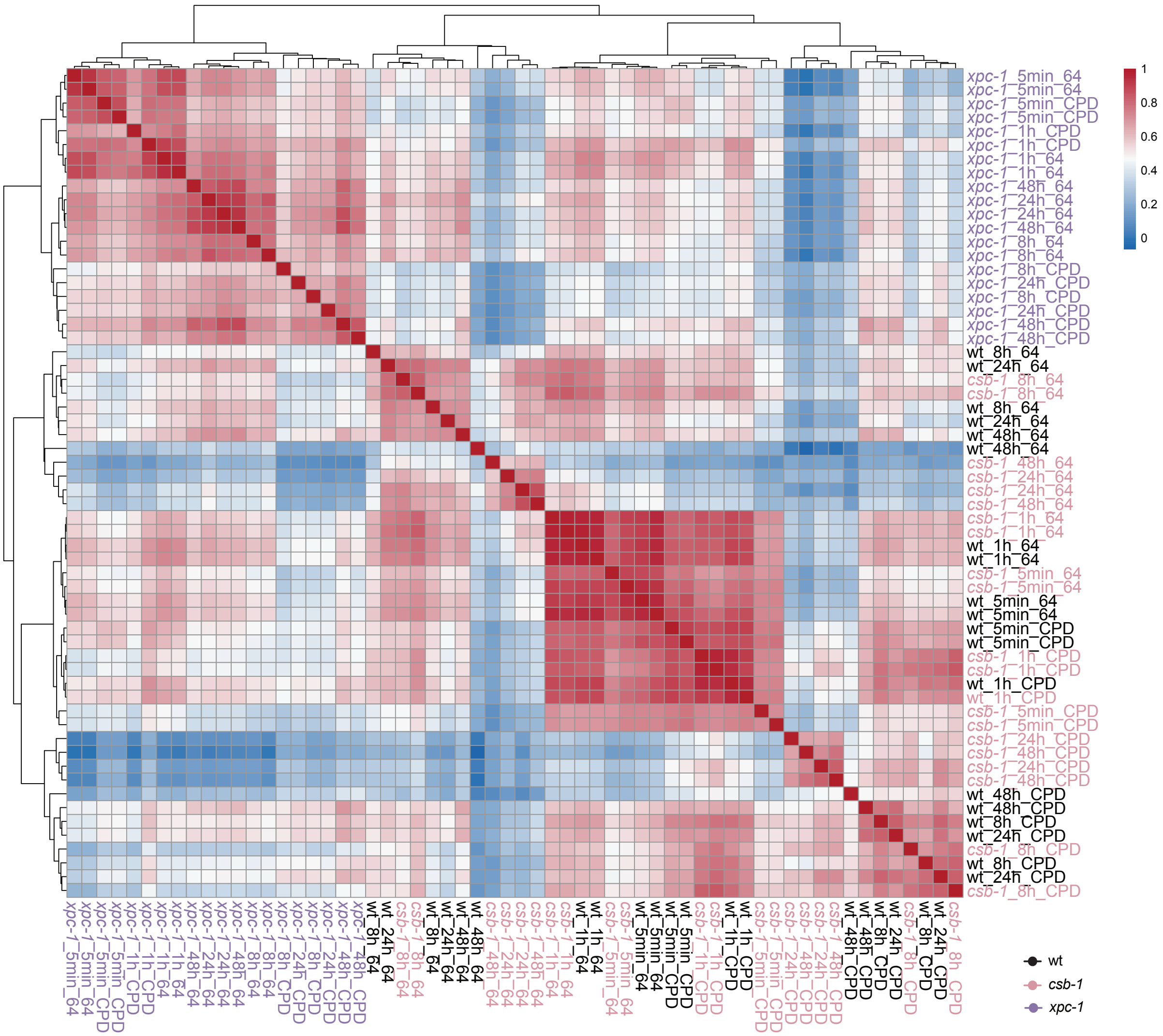

Supplementary Fig. 3: Hierarchical clustering of genome-wide repair profiles across biological replicates corresponding to the merged datasets presented in Fig. 2E. Heatmap illustrating pairwise Spearman correlations between XR-seq biological replicates from wild-type, *csb-1*, and *xpc-1*, calculated from RPM values across 2-kb genomic bins. Data are shown separately for each UV repair time point and damage type.

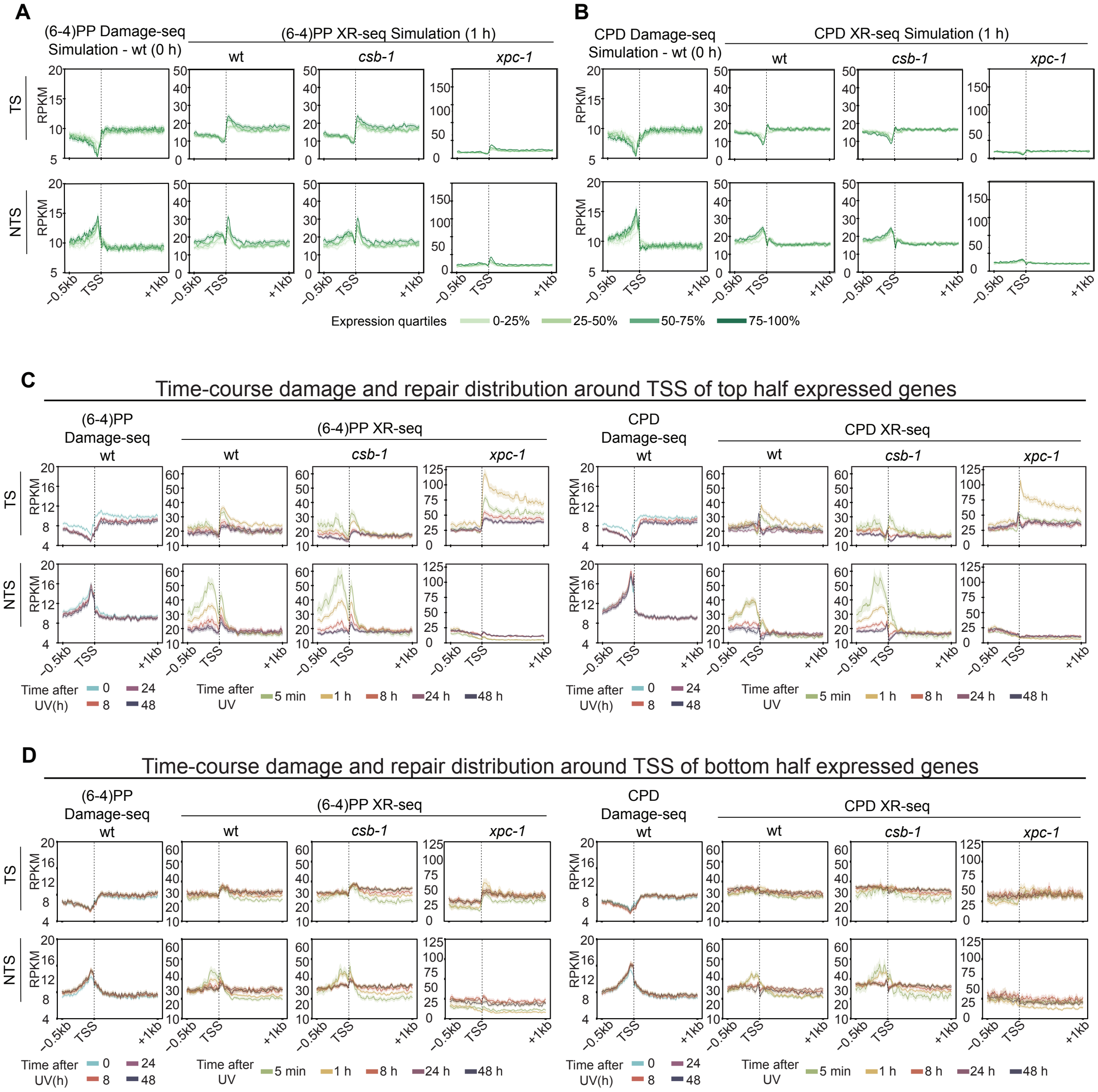

Supplementary Fig. 4: Simulated damage formation across gene expression quartiles and time-course profiles of Damage-seq and XR-seq signal at lowly expressed genes. A, B Simulated sequence-based predictions of (6-4)PP (A) and CPD (B) 0 h Damage-seq, and 1 h XR-seq data corresponding to the observed profiles shown in Fig. 3C–D. **C, D** Time-course line plots showing RPKM-normalized signal for (6-4)PP and CPD. Damage-seq signals are shown at 0 h, 8 h, 24 h, and 48 h post-UV; XR-seq signals are shown for wild-type (wt), *csb-1*, and *xpc-1* at 5 min, 1 h, 8 h, 24 h, and 48 h post-UV. Plots are centered on the transcription start sites (TSSs) of the top 50% (C) and bottom 50% (D) of expressed genes and span 500 bp upstream to 1 kb downstream. Signals are separated by transcribed strand (TS, top panels) and non-transcribed strand (NTS, bottom panels). Shaded areas represent 95% confidence intervals.

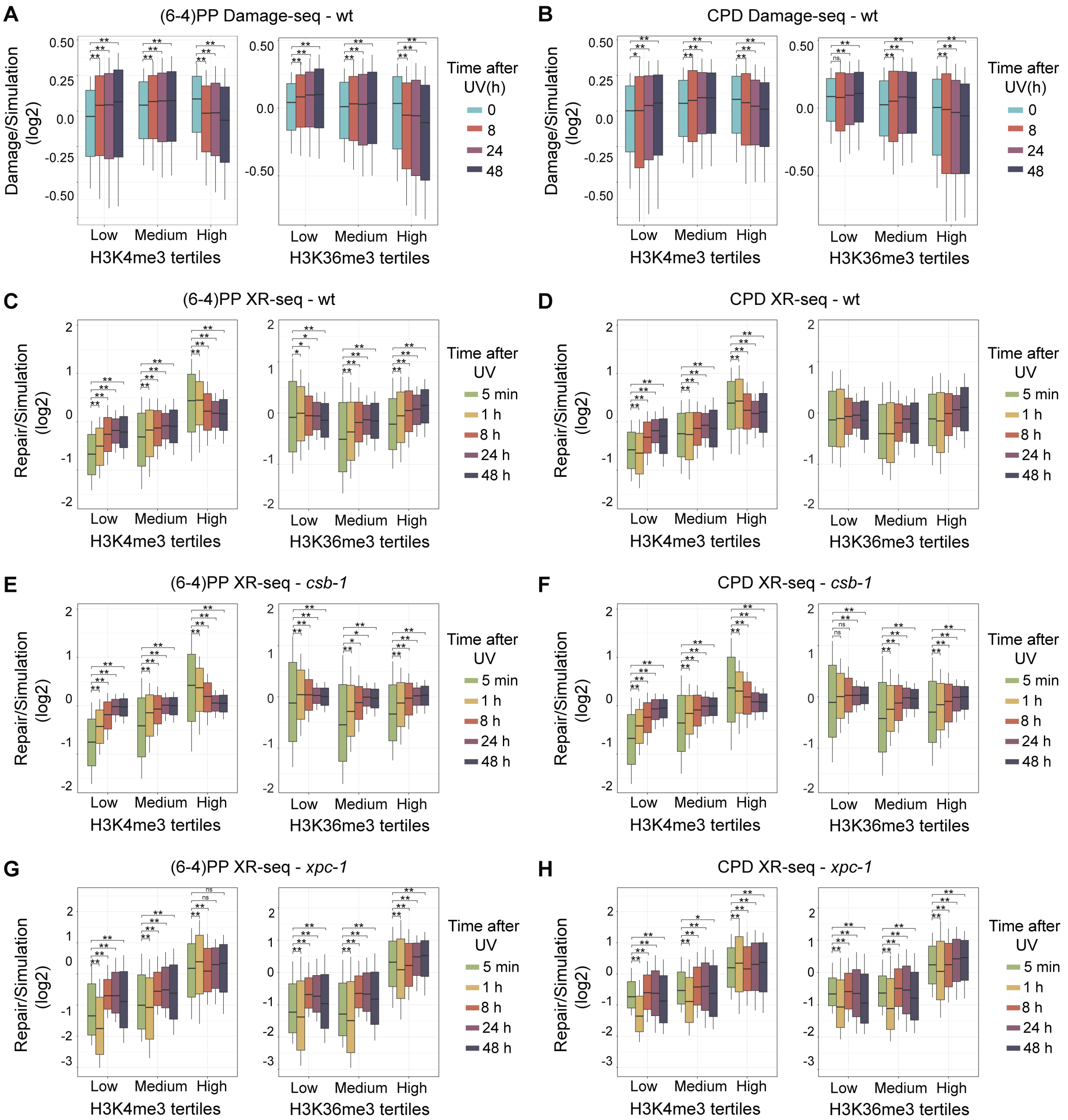

Supplementary Fig. 5: Time-course DNA damage and repair signals stratified by H3K4me3 and H3K36me3 tertiles. Boxplots show log_2-normalized Damage-seq (**A**–**B**) and XR-seq (**C**–**H**) signals divided by simulation profiles, grouped by tertiles of H3K4me3 (left) or H3K36me3 (right) signal in 2 kb bins. Data are shown for wild-type (A–D), *csb-1* (E–F), and *xpc-1* (G–H). Time points correspond to 0–48 h after UV irradiation for Damage-seq (A–B) and 5 min–48 h for XR-seq (C–H). Significance was determined by paired Wilcoxon tests relative to 5 min (within time course); stars denote adjusted *p*-values (BH-corrected). ns, not significant ($p \geq 0.05$); * $p < 0.05$; ** $p < 0.01$.

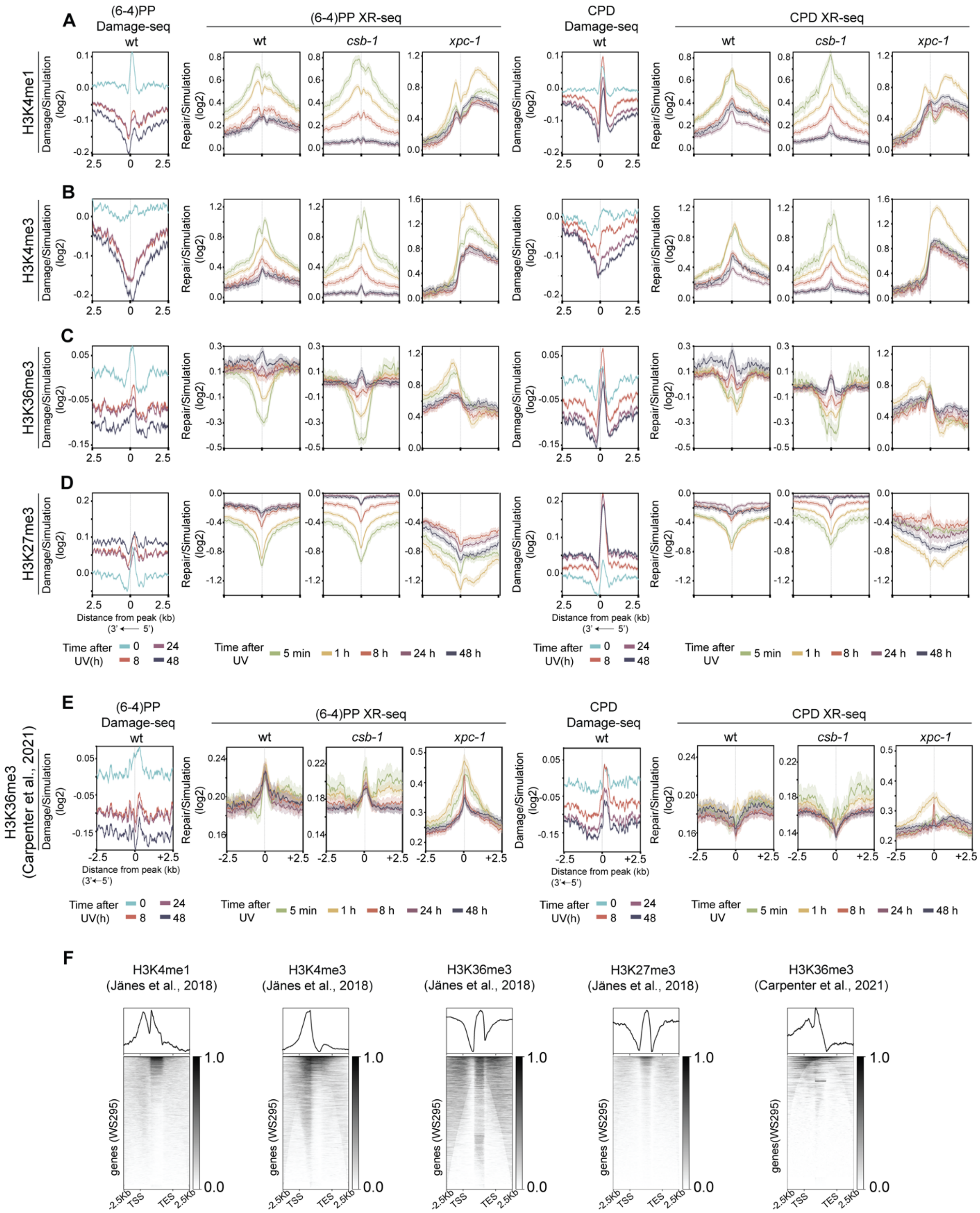

Supplementary Fig. 6: Distribution of DNA damage and repair around histone modification peaks. (**A**–**D**) Strand-specific line plots of $\log_2$(real/simulation) ratios centered on the same peak sets (±2.5 kb). Left subpanels show wt Damage-seq (0, 8, 24, 48 h); right subpanels show XR-seq (wt, *csb-1, xpc-1*; 5 min, 1, 8, 24, 48 h). (A) H3K4me1, (B) H3K4me3, (C) H3K36me3, (D) H3K27me3. Shaded areas denote 95% CIs. See Methods (“Strand assignment and orientation”) for details. **E** Damage-seq

and XR-seq time-course data shown in panel (C) were replotted using H3K36me3 ChIP-seq peak centers from Carpenter et al. (2021) instead of Jänes et al. (2018), to assess how differences in ChIP-seq datasets influence apparent repair and damage distributions. **F** Distribution of ChIP-seq peaks from Jänes et al. (2018) and Carpenter et al. (2021), visualized as heatmaps and corresponding aggregate line plots. Genes were pseudo-scaled to 1 kb with ±2.5 kb flanks. Heatmaps show peak density along gene bodies but do not reflect signal intensity.

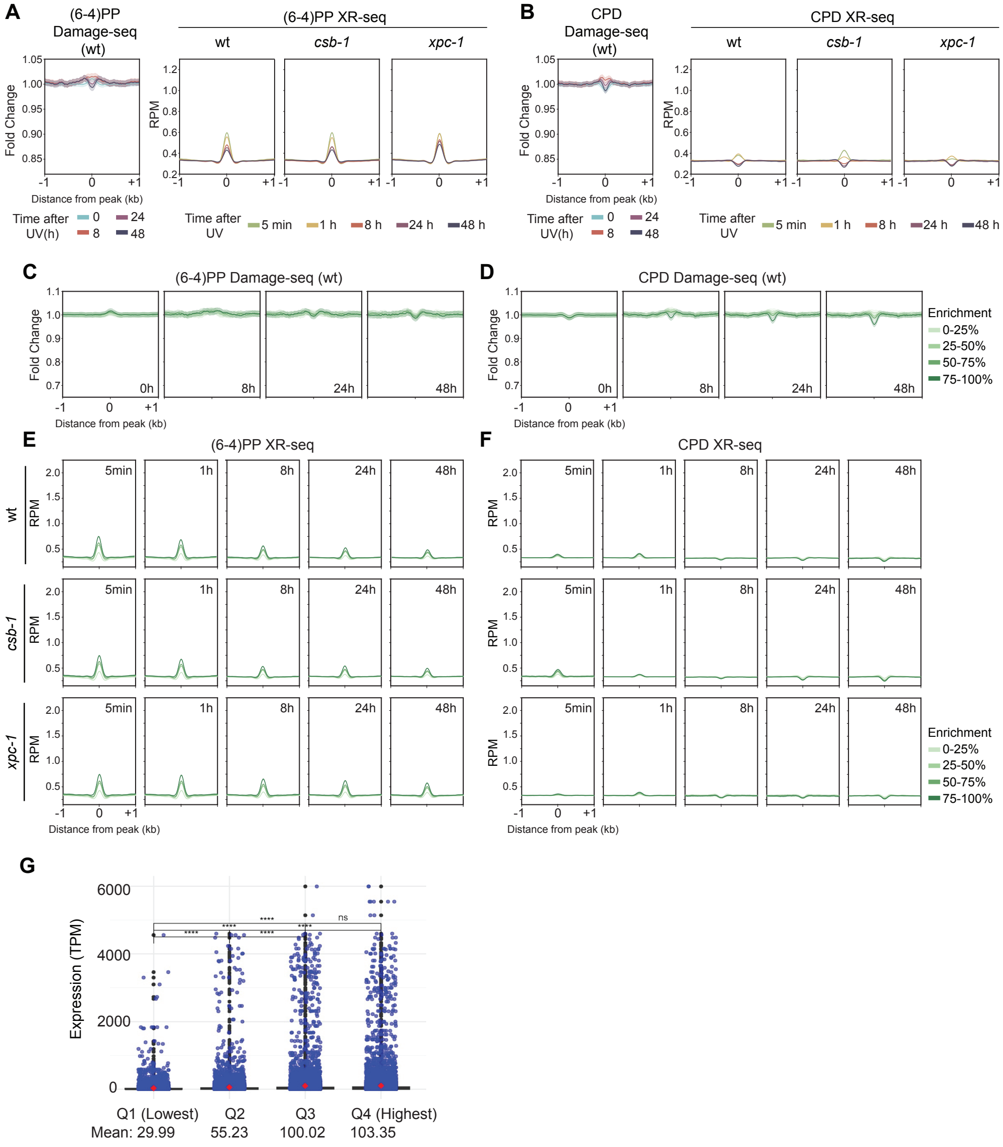

Supplementary Fig. 7: Simulation of damage and repair distribution around ATAC-seq peaks. A Line plots showing simulated Damage-seq (left panels) and XR-seq (right panels) signal for (6-4)PP and (**B**) CPD across ATAC-seq peak summits (±1 kb). Simulations of Damage-seq is for 0 h, 8 h, 24 h, and 48 h Damage-seq data; simulations of XR-seq is shown as RPM for wild-type (wt), *csb-1,* and *xpc-1* across 5 min, 1 h, 8 h, 24 h, and 48 h post-UV. **C** Simulated Damage-seq fold-change signal over time for (6-4)PP and (**D**) CPD, with ATAC-seq peaks stratified into four chromatin accessibility quartiles (0–25%, 25–50%, 50–75%, 75–100%; light to dark green). **E** Simulated XR-seq RPM signal over the same peak quartiles and time course for (6-4)PP and (**F**) CPD in wt, *csb-1*, and *xpc-1*. Shaded areas represent 95% confidence intervals. **G** Boxplot showing transcript per million (TPM) values for genes overlapping ATAC-seq peaks stratified into four quartiles. Each dot represents a gene, and red dots indicate the mean expression per quartile. Q1 includes the least accessible regions, while Q4 represents the most accessible. Mean TPM values are indicated below each quartile. Statistical comparisons between quartiles were performed using a two-sided Wilcoxon test; ****p < 0.0001, ns = not significant.

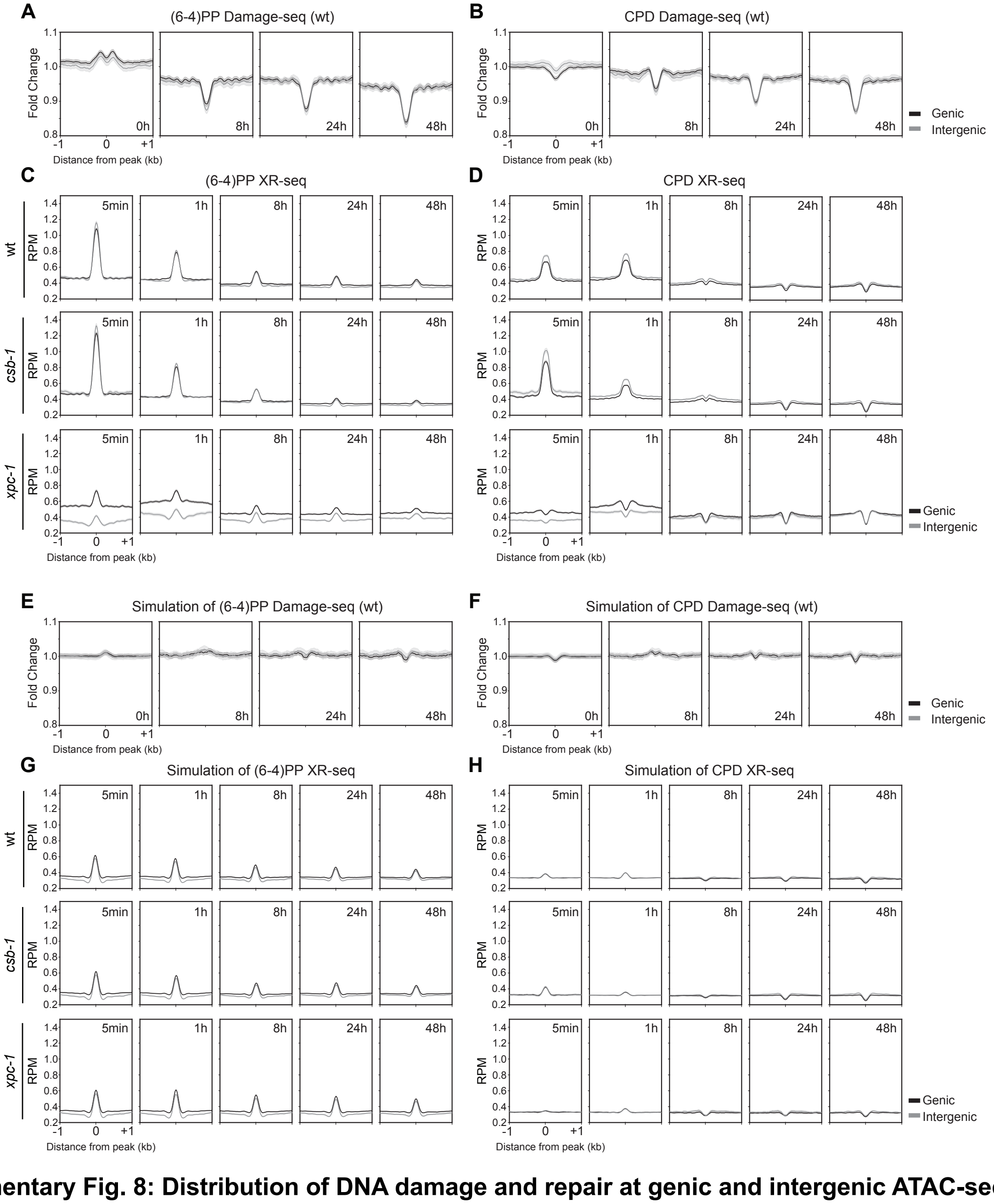

Supplementary Fig. 8: Distribution of DNA damage and repair at genic and intergenic ATAC-seq peaks. A Line plots show time-course Damage-seq signal for (6-4)PP and (**B**) CPD at ATAC-seq peak centers (±1 kb). Signals are normalized to both input and naked DNA and plotted as fold change. ATAC-seq peaks were categorized as either genic (black) or intergenic (grey) based on overlap with gene annotations. **C** XR-seq signal (reads per million, RPM) for (6-4)PP and (**D**) CPD is plotted for wild-type (wt), *csb-1*, and *xpc-1* across the same genic and intergenic peak sets and over the UV repair time course (5 min to 48 h). **E** Simulated Damage-seq signal for (6-4)PP and (**F**) CPD over the same peak sets and time points. **G** Simulated XR-seq signal for (6-4)PP and (**H**) CPD in wt, *csb-1*, and *xpc-1*, plotted as RPM across genic and intergenic peaks. Shaded regions represent 95% confidence intervals.

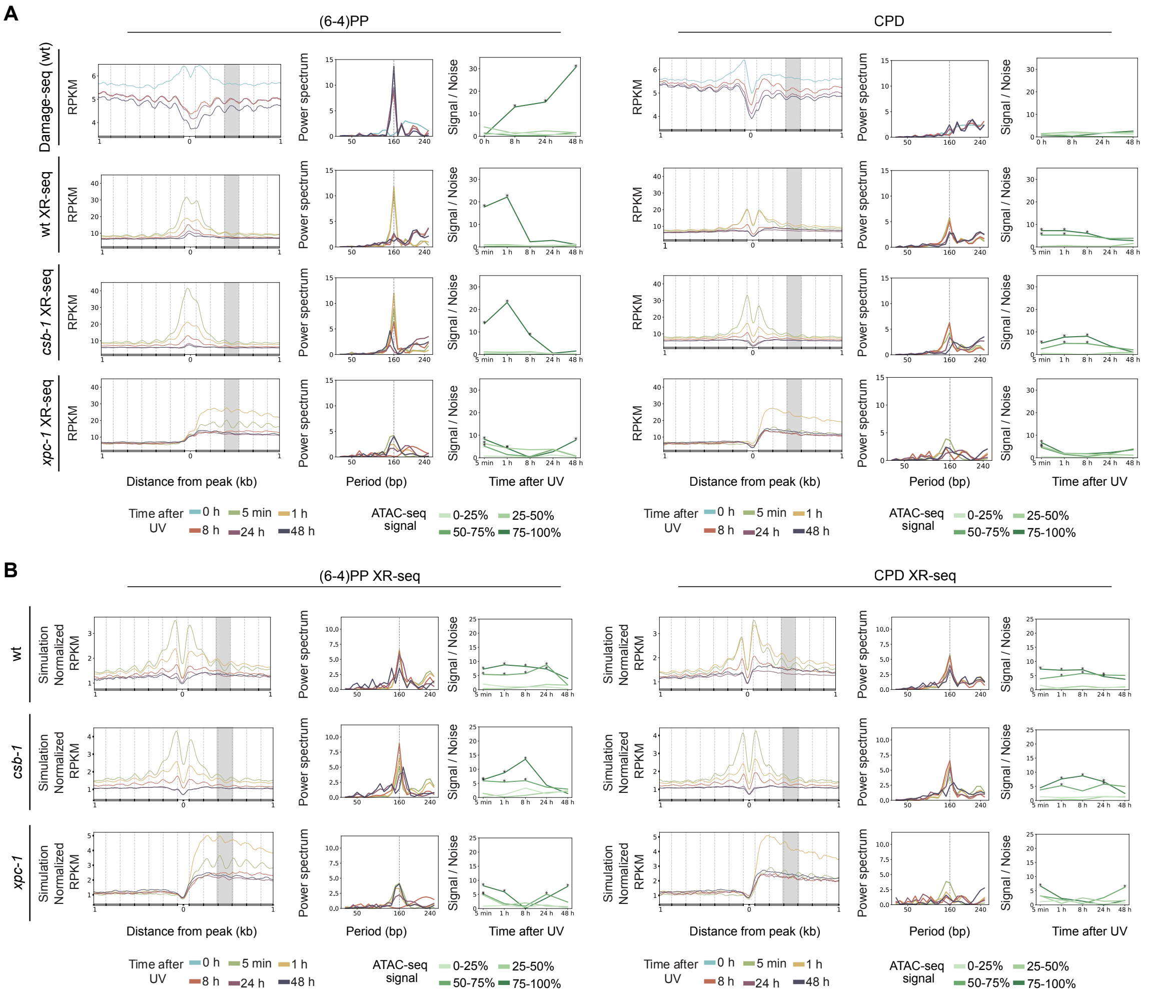

Supplementary Fig. 9: A Line plots of Damage-seq and XR-seq signals shown as raw RPKM values for (6-4)PP (left) and CPD (right), centered on the most accessible quartile of L1-stage ATAC-seq peaks across time points. Damage-seq signals are shown at 0 h, 8 h, 24 h, and 48 h post-UV, while XR-seq signals are shown for wt, *csb-1*, and *xpc-1* mutants at 5 min, 1 h, 8 h, 24 h, and 48 h post-UV. Power spectrum and signal-to-noise ratio (SNR) plots quantify 160 bp periodicity in observed Damage-seq and XR-seq signals across time points. **B** Simulation-normalized XR-seq signals for (6-4)PP (left) and CPD (right), plotted across the same time points and peak centers. Power spectrum and SNR plots quantify 160 bp periodicity in normalized signals. Gray bars below each plot indicate nucleosome-wrapped regions, and black bars indicate linker DNA positions. Highlighted areas show profiles at the third nucleosome position relative to ATAC-seq peak centers. All panels span ±1 kb from the ATAC-seq peak center.

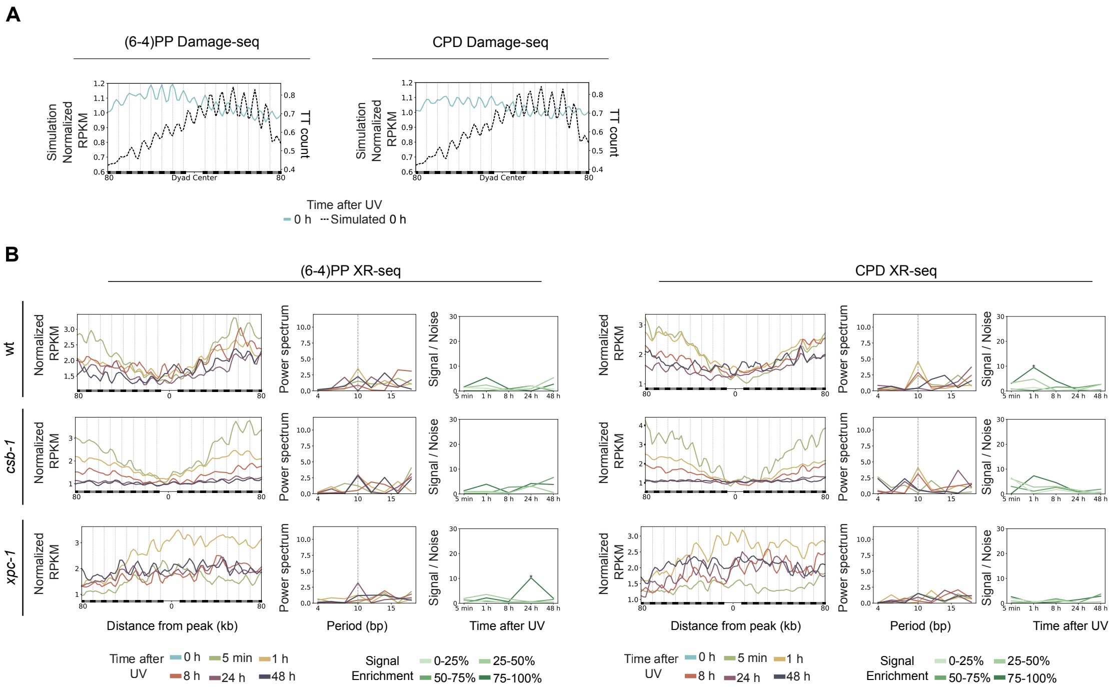

Supplementary Figure 10: Periodicity of UV-induced simulation-normalized damage and repair signals, and simulation- and damage-normalized repair signals centered on dyad positions. A Simulation-normalized Damage-seq signals for (6-4)PP (left) and CPD (right) at 0 h post-UV, centered on nucleosome dyad positions and plotted against TT dinucleotide counts to assess sequence context contributions to ~10 bp periodicity. **B** Damage- and simulation-normalized XR-seq signals for (6-4)PP (left) and CPD (right) across the same time points, with corresponding power spectrum and SNR plots quantifying 10 bp periodicity. Gray and black bars below each plot indicate minor-in and minor-out positions, respectively. All panels span ±80 bp from the dyad center.
